# Three distinct mechanisms, Notch instructive, permissive, and independent, regulate the expression of two different pericardial genes to specify cardiac cell subtypes

**DOI:** 10.1371/journal.pone.0241191

**Published:** 2020-10-27

**Authors:** Manoj Panta, Andrew J. Kump, John M. Dalloul, Kristopher R. Schwab, Shaad M. Ahmad

**Affiliations:** 1 Department of Biology, Indiana State University, Terre Haute, Indiana, United States of America; 2 The Center for Genomic Advocacy, Indiana State University, Terre Haute, Indiana, United States of America; 3 Terre Haute South Vigo High School, Terre Haute, Indiana, United States of America; 4 Stanford University, Stanford, California, United States of America; National Institutes of Health, UNITED STATES

## Abstract

The development of a complex organ involves the specification and differentiation of diverse cell types constituting that organ. Two major cell subtypes, contractile cardial cells (CCs) and nephrocytic pericardial cells (PCs), comprise the *Drosophila* heart. Binding sites for Suppressor of Hairless [Su(H)], an integral transcription factor in the Notch signaling pathway, are enriched in the enhancers of PC-specific genes. Here we show three distinct mechanisms regulating the expression of two different PC-specific genes, *Holes in muscle* (*Him*), and *Zn finger homeodomain 1* (*zfh1*). *Him* transcription is activated in PCs in a permissive manner by Notch signaling: in the absence of Notch signaling, Su(H) forms a repressor complex with co-repressors and binds to the *Him* enhancer, repressing its transcription; upon alleviation of this repression by Notch signaling, *Him* transcription is activated. In contrast, *zfh1* is transcribed by a Notch-instructive mechanism in most PCs, where mere alleviation of repression by preventing the binding of Su(H)-co-repressor complex is not sufficient to activate transcription. Our results suggest that upon activation of Notch signaling, the Notch intracellular domain associates with Su(H) to form an activator complex that binds to the *zfh1* enhancer, and that this activator complex is necessary for bringing about *zfh1* transcription in these PCs. Finally, a third, Notch-independent mechanism activates *zfh1* transcription in the remaining, *even skipped*-expressing, PCs. Collectively, our data show how the same feature, enrichment of Su(H) binding sites in PC-specific gene enhancers, is utilized by two very distinct mechanisms, one permissive, the other instructive, to contribute to the same overall goal: the specification and differentiation of a cardiac cell subtype by activation of the pericardial gene program. Furthermore, our results demonstrate that the *zfh1* enhancer drives expression in two different domains using distinct Notch-instructive and Notch-independent mechanisms.

## Introduction

The remarkable cellular diversity observed in metazoan organs raises an important developmental question: how are the specification and differentiation of the many distinct cell types comprising such an organ achieved?

**Fig 1:**
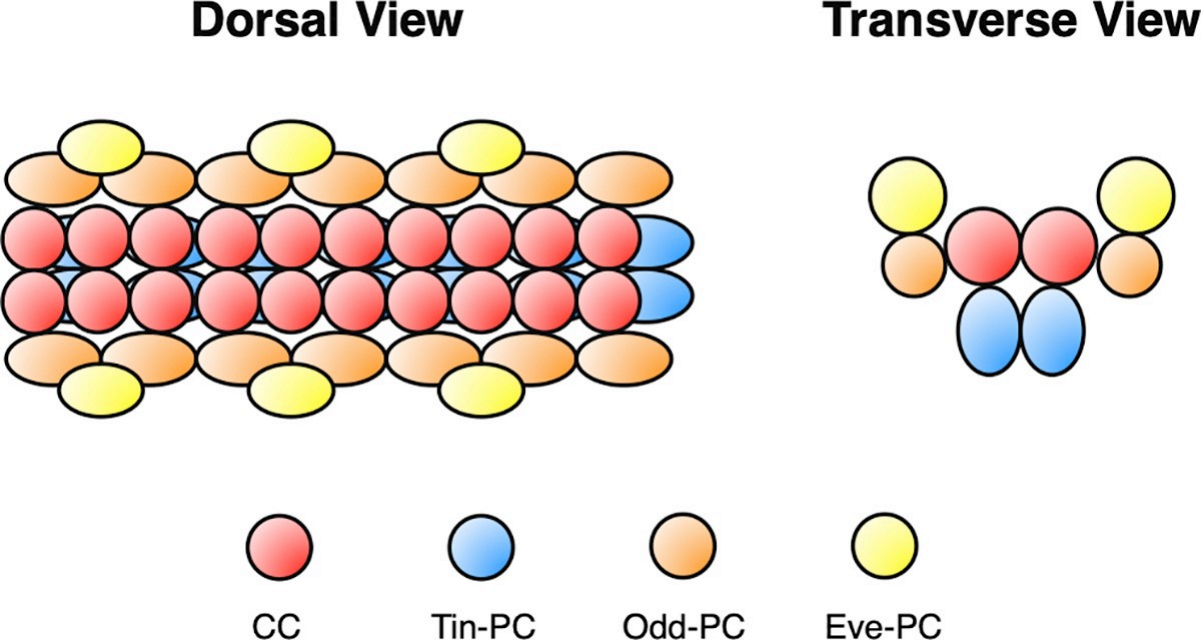
Stereotyped positions of cardiac cell subtypes. Schematic diagram showing the relative positions of the nuclei of cardial cells (CCs) and three classes of pericardial cells (PCs): Tin-PCs, Odd-PCs, and Eve-PCs.

The embryonic *Drosophila* heart provides a particularly amenable system for addressing this question. An organ that pumps hemolymph throughout the body cavity, the *Drosophila* heart consists of two groups of cells: an inner linear tube of paired contractile cardial cells (CCs) that express *Myocyte enhancer factor 2* (*Mef2*), and an external sheath of nephrocytic pericardial cells (PCs) that express both *Zn finger homeodomain 1* (*zfh1*) and *Holes in muscle* (*Him*) [1–4]. Neither the CCs nor the PCs constitute a uniform population. In particular, the PCs can be subdivided into additional cell subtypes based on their positions, the complexity of their individual gene expression programs, their morphology, and even the distinct cell lineages that generate them [5]. A paired row of PCs expressing *tinman* (*tin*) and referred to as Tin-PCs runs immediately ventral to the CC tube; PCs expressing *odd-skipped* (*odd*) and referred to as Odd-PCs are positioned laterally with respect to the CCs; and PCs expressing both *even-skipped* (*eve*) and *tin*, referred to as Eve-PCs, are located dorsolaterally (Fig 1) [6–9].

Enhancers, stretches of DNA that are recognized and bound by particular combinations of sequence-specific DNA binding transcription factors (TFs), determine gene expression programs at the level of individual cells [10]. Previously, using a machine learning approach to identify the enhancers and binding motifs which determine the expression patterns of genes expressed in specific cardiac cell subtypes, we found that motifs corresponding to binding sites for Su(H), a transcription factor (TF) in the Notch signaling pathway [11–14], were enriched in the enhancers of PC-specific genes [3]. Further analysis of the enhancer of the PC-specific gene *Him* suggested that Su(H) forms a repressor complex that binds to this enhancer, with Notch signaling alleviating the repression in PCs to activate *Him* transcription [3].

Here we present additional evidence indicating that Su(H) forms a repressor complex to prevent transcription from the *Him* enhancer in CCs and show further that the mechanisms by which the gene *zfh1* is expressed specifically in PCs are completely distinct from that used for *Him*. Our results suggest that Su(H) associates with the Notch intracellular domain (N^icd^) to form an activator complex whose binding to the *zfh1* enhancer is essential for *zfh1* transcription in the Odd-PCs and Tin-PCs; mere alleviation of Su(H)-mediated repression is not sufficient as it was in the case of *Him*. Moreover, transcription from the same *zfh1* enhancer in Eve-PCs is mediated by a third pathway independent of both Su(H) and Notch signaling. Our results illustrate how the same feature, enrichment of Su(H) binding sites in PC-specific *Him* and *zfh1* gene enhancers, is utilized in two distinct ways to achieve the same overall goal, the activation of the pericardial gene program. Additionally, we demonstrate how one particular enhancer, that of *zfh1*, is regulated in two different ways to produce the same result, transcriptional activation, in different pericardial subsets.

## Materials and methods

### Transgenic reporter constructs

Su(H) binding sites were identified in the *Him* and *zfh1* enhancers by scanning their sequences for any matches to YGTGDGAA but not TGTGTGAA as reported previously [14–18]. Both wild-type enhancer regions (sequences in S1 and S2 Files), as well as *Him* and *zfh1* enhancer regions with single base-pair mutations at these binding sites to eliminate Su(H) binding (S3 and S4 Files) [15], were synthesized *in vitro* (Integrated DNA Technologies, Coralville, IA, USA), sequence verified, and subcloned into *pWattB-nlacZ* [3, 19, 20]. Details of this cloning vector are presented in S1 Fig and its complete sequence is available from GenBank with the accession number MT747949 and in S6 File. The resulting reporter constructs were targeted to the *attP40* docking site with phiC31-mediated integration [21] (GenetiVision, Houston, TX, USA) to create lines with wild-type enhancers driving β-galactosidase reporters (*Him*^*WT*^*-lacZ* and *zfh1*^*WT*^*-lacZ*) as well as lines with reporters driven by enhancers lacking functional Su(H) binding sites (*Him*^*Su(H)*^*-lacZ* and *zfh1*^*Su(H)*^*-lacZ*).

### *Drosophila* strains and genetics

*Drosophila* stocks containing the following transgenes were used: *attP40* (FlyBase ID: FBti0114379) [21], *Su(H)*^*HMS05748*^ (FlyBase ID: FBal0326016) [22, 23], and *Su(H)*^*HM05110*^ (FlyBase ID: FBal0240240) [22, 23], *N*^*HMS00001*^ (FlyBase ID: FBal0257052) [22, 23], *N*^*HMS00009*^ (FlyBase ID: FBal0257057) [22, 23], *gro*^*KK108953*^ (FlyBase ID: FBal0259754) [24], *H*^*GD1458*^ (FlyBase ID: FBal0207248) [24], *CtBP*^*KK108401*^ (FlyBase ID: FBal0235549) [24], *eve*^*HMS01312*^ (FlyBase ID: FBal0257905) [22, 23], *UAS-Dcr-2* (*Dcr-2*^*UAS*.*cDa*^; FlyBase ID: FBal0211026) [24], *UAS-N*^*icd*^ (*N*^*int*.*SH*.*UAS*^; FlyBase ID: FBal0093233) [25, 26], *TinD-GAL4* (*GAL4*^*tin*.*D*^; FlyBase ID: FBal0267971) [27], *Hand-GAL4* (*GAL4*^*HCH*.*Hand*^; FlyBase ID: FBal0267971) [28], *Him*^*WT*^*-lacZ* (this study), *Him*^*Su(H)*^*-lacZ* (this study), *zfh1*^*WT*^*-lacZ* (this study), and *zfh1*^*Su(H)*^*-lacZ* (this study). *Su(H)*^*HMS05748*^, *Su(H)*^*HM05110*^, *N*^*HMS00001*^, *N*^*HMS00009*^, *eve*^*HMS01312*^ and *Dcr-2*^*UAS*.*cDa*^ were obtained from the Bloomington Drosophila Stock Center; *gro*^*KK108953*^, *H*^*GD1458*^, and *CtBP*^*KK108401*^ were obtained from the Vienna Drosophila Resource Center; *GAL4*^*tin*.*D*^ was a gift from A. Michelson; *GAL4*^*HCH*.*Hand*^ was gift from Z. Han; and *N*^*int*.*SH*.*UAS*^ was a gift from M. Halfon.

The complete genotypes of the embryos used to examine activity from transgenic reporter constructs via fluorescent immunohistochemistry, the number of embryos examined for each genotype as well as the number of embryos exhibiting specific phenotypes, and a list of the figures showing representative embryos of each genotype are provided in S1 Table.

### RNA interference assays

RNA interference (RNAi) was performed by using *TinD-GAL4* to drive expression from relevant UAS-RNAi constructs specifically in the cardiac mesoderm of embryos also carrying the appropriate enhancer-reporter construct (S1 Table). Representative images of reporter activity from control embryos possessing only the *TinD-GAL4* driver or the UAS-RNAi constructs alone are presented in S2 and S3 Figs. The efficiency of RNA interference (RNAi) knockdowns was enhanced by allowing embryos to develop at 29°C. Reverse transcription quantitative real-time PCR (RT-qPCR) from total RNA isolated from stage 13–16 embryos was used to assess the efficacy of RNAi knockdowns with the methodology, primers, and results presented in S5 File. Note that the fact that considerable reduction in gene product levels of the relevant genes is detected with knockdowns while using RNA from whole embryos despite the knockdown occurring in only a minute subset of embryonic cells (the cardiac mesoderm) indicates that the RNAi-induced knockdown in the cardiac mesoderm is quite significant.

### GAL4 drivers

Two distinct GAL4 drivers were used in this study. The *TinD-GAL4* driver that was used for both the RNAi knockdowns and ectopic expression of *N*^*icd*^ is active between embryonic stages 11 and 12 in the cardiac mesoderm as it is being specified and a little later [27]. The *Hand-GAL4* driver that was used only for ectopic expression of *N*^*icd*^ is active in the developing heart from late embryonic stage 12 to the end of embryogenesis [28]. Both GAL4 drivers were tested with *UAS-lacZ* reporters to ensure that they drove expression in every cardiac mesoderm and heart cell (S4 Fig)

### Immunohistochemistry

Whole embryo fluorescent immunohistochemistry followed standard protocols [3, 20]. The following primary antibodies were used: rabbit anti-Mef2 (1:1000, gift from B. Paterson; 1:7500, gift from J. Jacobs) [29, 30], guinea pig anti-Zfh1 (1:1000, gift from J. Skeath) [31], guinea pig anti-Eve (1:100, gift from D. Kosman and J. Reinitz) [32], rat anti-Odd (1:100, gift from D. Kosman and J. Reinitz) [32] and mouse anti-β–galactosidase (1:500, Promega, Madison, WI, USA).

## Results

### Su(H) represses transcription mediated by the *Him* enhancer in cardial cells

**Fig 2:**
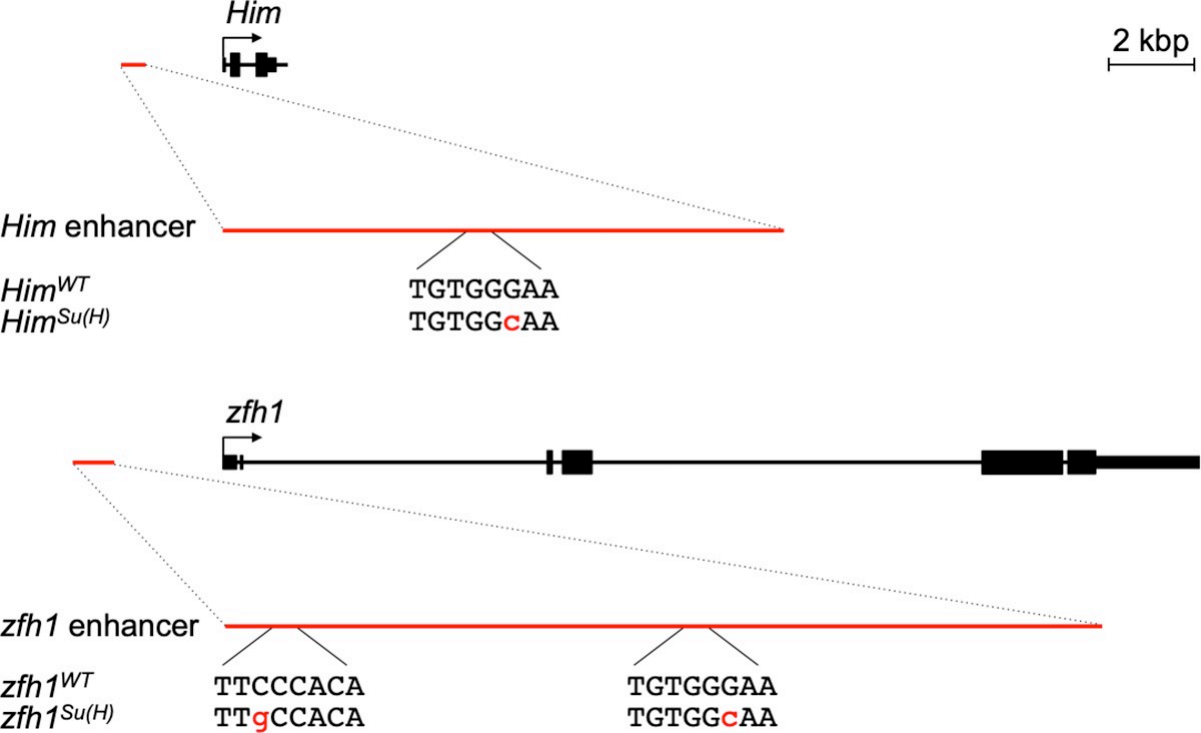
Su(H) binding sites in the *Him* and *zfh1* enhancers. Both the *Him* enhancer (X:18,206,943..18,207,537) and the *zfh1* enhancer (3R:30,762,904..30,763,833) are located 5´ to the endogenous genes (*Drosophila melanogaster* genome sequence release 6.29). The relative locations of the Su(H) binding sites on these enhancers are shown, as well as their wild-type sequences (uppercase). The nucleotide substitutions used to create binding site mutations that eliminate Su(H) binding are shown in red lowercase. Sequences for the wild-type and mutated enhancers are provided in S1–S4 Files.

**Fig 3:**
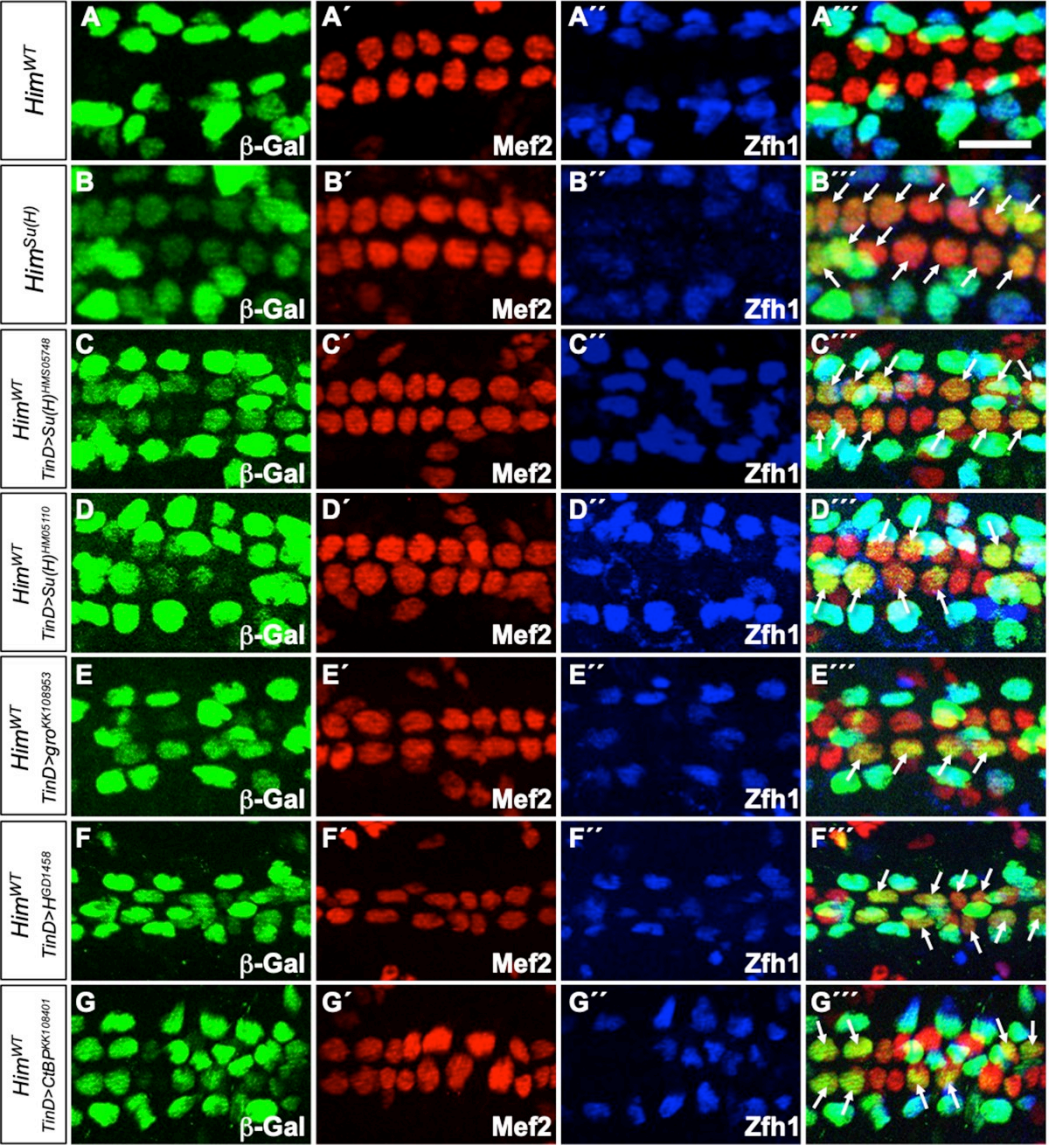
Su(H), Groucho, Hairless, and CtBP function as repressors for the *Him* enhancer in CCs. (A-G´´´) *lacZ* reporter gene activity (β–galactosidase, green) driven by relevant *Him* enhancers in appropriate genotypes of stage 16 embryos. All CCs express Mef2 (red) while PCs are marked by Zfh1 (blue). Scale bar: 10 μm. (A-A´´´) The wild-type *Him* enhancer (*Him*^*WT*^) is active only in the Zfh1-expressing PCs. (B-B´´´) When Su(H) binding sites are mutated in the *Him* enhancer (*Him*^*Su(H)*^), the reporter is still active in Zfh1-expressing PCs but is also de-repressed in Mef2-expressing CCs (arrows). (C-C´´´) Knockdown of Su(H) with the dorsal mesoderm-targeted RNAi construct *Su(H)*^*HMS05748*^ driven by the *TinD-GAL4* driver induces ectopic *Him*^*WT*^ enhancer-driven β–galactosidase reporter activity in CCs (arrows). The fact that not all CCs express the same level of ectopic reporter activity likely reflects an incomplete RNAi knockdown of Su(H) levels in all cardiac cells. (D-D´´´) Similar ectopic *Him*^*WT*^ enhancer-driven β–galactosidase reporter activity in CCs (arrows) is also induced by the *TinD-GAL4* driven knockdown of Su(H) with a second RNAi construct, *Su(H)*^*HM05110*^. (E-G´´´) Dorsal mesoderm targeted knockdown of the co-repressors Groucho (E-E´´´), Hairless (F-F´´´), and CtBP (G-G´´´) by the *TinD-GAL4* driven expression of the constructs *gro*^*KK108953*^, *H*^*GD1458*^, and *CtBP*^*KK108401*^, respectively, also result in the ectopic induction of *Him*^*WT*^ enhancer-driven β–galactosidase reporter activity in CCs (arrows) similar to that detected when either the Su(H) binding site is mutated in the enhancer (B-B´´´) or Su(H) is knocked down (C-D´´´). Sample sizes for these assays can be found in S1 Table.

We had previously identified a 595 bp *Him* enhancer (*Him*^*WT*^) and a 930 bp *zfh1* enhancer (*zfh1*^*WT*^) with one and two Su(H) binding sites, respectively (Fig 2; sequences in S1 and S2 Files), that recapitulated the PC-specific expression of endogenous *Him* and *zfh1* with β–galactosidase reporters in all three PC subtypes: Tin-PCs, Odd-PCs, and Eve-PCs (Figs 3A–3A´´´; 4A and 4A; Fig 5A and 5A, 5E–5E´´´) [3]. As we had already shown, abolishing Su(H) binding to the *Him* enhancer by mutating the Su(H) binding site (*Him*^*Su(H)*^) (Fig 2; sequence in S3 File) induces ectopic reporter activity in all of the CCs without affecting expression in PCs (Fig 3B–3B´´´; S1 Table) [3]. Similar ectopic reporter activity in the CCs mediated by the wild-type *Him* enhancer (*Him*^*WT*^) is also observed when *Su(H)* levels are depleted by RNA interference (RNAi) knockdowns driven by the cardiac mesoderm-specific *TinD-GAL4* driver (Fig 3C–3D´´´; S1 Table; S4 Fig) [3]. Collectively, these observations indicate that Su(H) functions to repress *Him* activity in the cardial cells.

**Fig 4:**
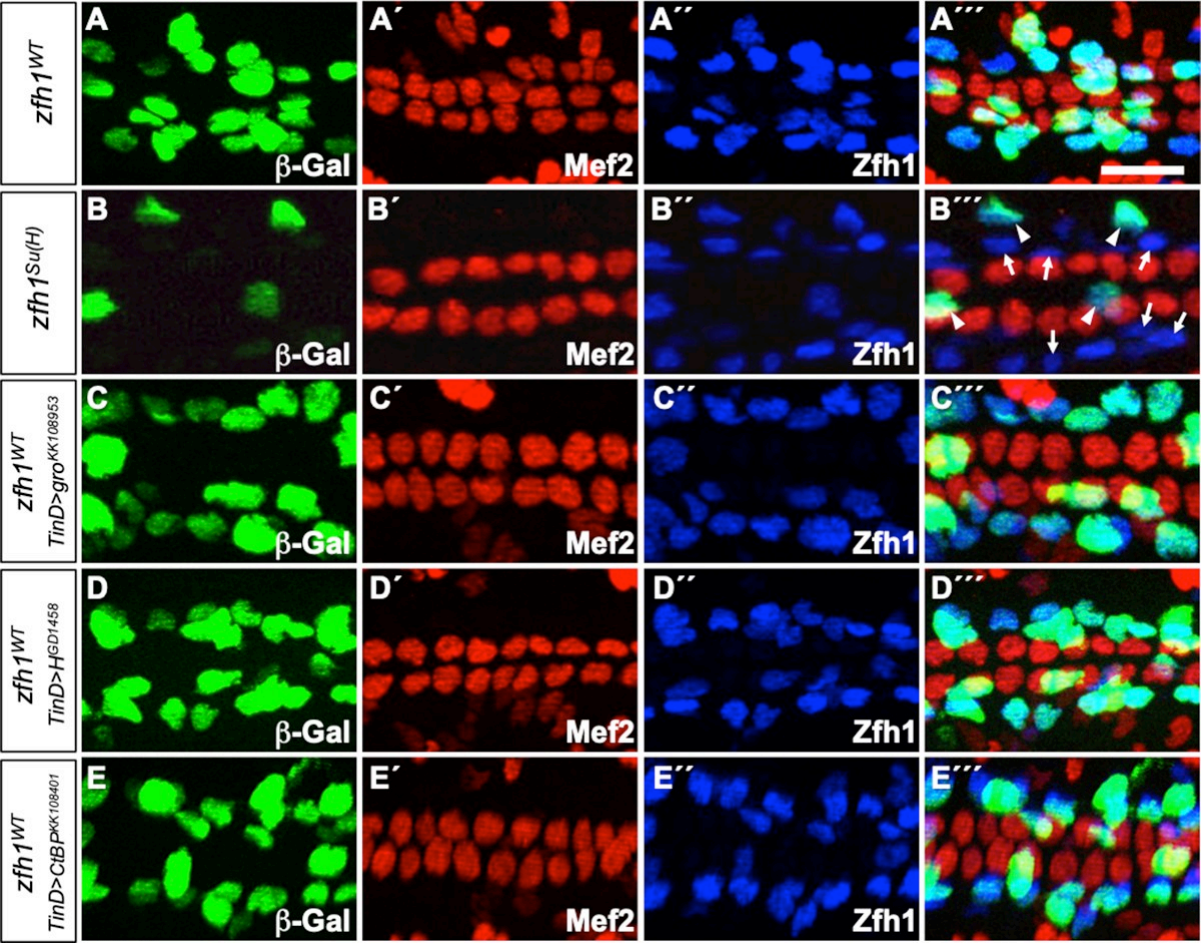
Su(H) functions as an activator for the *zfh1* enhancer in a subset of PCs while Groucho, Hairless, and CtBP have no effect on *zfh1* enhancer-driven expression. (A-E) *lacZ* reporter gene activity (β–galactosidase, green) driven by relevant *zfh1* enhancers in appropriate genotypes of stage 16 embryos. All CCs express Mef2 (red) while PCs are marked by Zfh1 (blue). Scale bar: 10 μm. (A-A´´´) The wild-type *zfh1* enhancer (*zfh1*^*WT*^) drives *lacZ* reporter expression exclusively in all PCs. (B-B´´´) Mutagenesis of Su(H) binding sites in the *zfh1* enhancer (*zfh1*^*Su(H)*^) abrogates reporter activity in Odd-PCs (arrows) and Tin-PCs (arrows) but not in Eve-PCs (arrowheads) compared to the wild-type enhancer (*zfh1*^*WT*^). The specific pericardial cell subtypes where expression was eliminated were initially identified based on their relative positions in the heart and subsequently confirmed (see Fig 5) by staining with PC subtype-specific antibodies. (C-E´´´) Depletion of Groucho (C-C´´´), Hairless (D-D´´´), or CtBP (E-E´´´) with cardiac mesoderm-targeted RNAi by the *TinD-GAL4* driven expression of the constructs *gro*^*KK108953*^, *H*^*GD1458*^, and *CtBP*^*KK108401*^, respectively, neither induced ectopic *zfh1*^*WT*^ enhancer-driven reporter activity in CCs nor eliminated reporter activity in any PCs.

**Fig 5:**
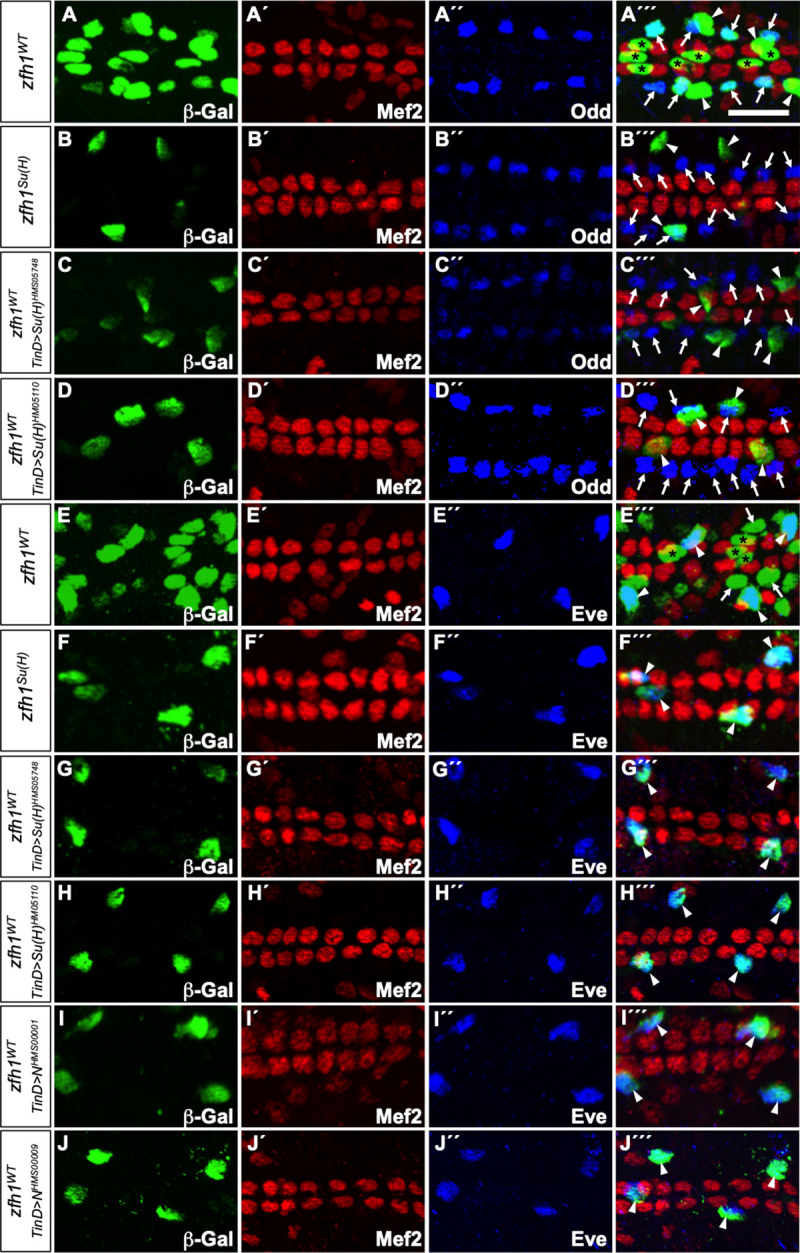
Su(H) and Notch activate expression from the *zfh1* enhancer in Odd-PCs and Tin-PCs. (A-J´´´) *lacZ* reporter gene activity (β–galactosidase, green) driven by relevant *zfh1* enhancers in appropriate genotypes of stage 16 embryos. Scale bar: 10 μm. All CCs express Mef2 (red) while in (A-D´´´) Odd-PCs are marked by Odd (blue), and in (E-J´´´) Eve-PCs are marked by Eve (blue). (A-A´´´) The wild-type *zfh1* enhancer (*zfh1*^*WT*^) is active in all PCs including the Odd-PCs (arrows), the ventrally located Tin-PCs (asterisks), and Eve-PCs (arrowheads). (B-B´´´) When Su(H) binding sites are mutated in the *zfh1* enhancer (*zfh1*^*Su(H)*^), reporter activity is only observed in Eve-PCs (arrowheads) and abrogated in Odd-PCs (arrows). The Eve-PC at the bottom of the panel is located dorsally above and masking an Odd-PC. Note that reporter expression is not detected in any ventral Tin-PCs either. (C-D´´´) Depletion of Su(H) with cardiac mesoderm-targeted RNAi using the constructs *Su(H)*^*HMS05748*^ (C-C´´´) and *Su(H)*^*HM05110*^ (D-D´´´) driven by the *TinD-GAL4* driver also maintains *zfh1*^*WT*^ enhancer-driven β-galactosidase reporter activity in Eve-PCs (arrowheads) and almost completely eliminates reporter expression in Odd-PCs (arrows) and Tin-PCs. The observation that some extremely faint reporter expression is detected in a few Tin-PCs and Odd-PCs likely reflects an incomplete RNAi knockdown of Su(H) levels in all heart cells. (E-E´´´) The wild-type *zfh1* enhancer (*zfh1*^*WT*^) is active in all PCs including the Eve-PCs (arrowheads), Odd-PCs (arrows), and Tin-PCs (asterisks). (F-F´´´) When Su(H) binding sites are mutated in the *zfh1* enhancer (*zfh1*^*Su(H)*^), the reporter is only expressed in Eve-PCs (arrowheads). (G-H´´´) Depletion of Su(H) with cardiac mesoderm-targeted RNAi using the constructs *Su(H)*^*HMS05748*^ (G-G´´´) and *Su(H)*^*HM05110*^ (H-H´´´) driven by the *TinD-GAL4* driver also eliminates *zfh1*^*WT*^ enhancer-driven β-galactosidase reporter activity in all PCs other than the Eve-PCs (arrowheads). (I-J´´´) Knockdown of Notch with cardiac mesoderm-targeted RNAi using the constructs *N*^*HMS00001*^ (I-I´´´) and *N*^*HMS00009*^ (J-J´´´) driven by the *TinD-GAL4* driver also eliminates *zfh1*^*WT*^ enhancer-driven reporter activity in all PCs other than the Eve-PCs (arrowheads).

### The co-repressors Groucho, Hairless, and C-terminal binding protein also repress transcription mediated by the *Him* enhancer in cardial cells

Since Su(H) had previously been shown to form transcriptional repressor complexes with the co-repressors Groucho (Gro), Hairless (H), and C-terminal Binding Protein (CtBP) [33–35], we examined the effects of knocking down the genes for each of these proteins. If Su(H) was forming a complex with these co-repressors to repress transcription from the *Him* enhancer in CCs, then a knockdown of any one of these co-repressors should also recapitulate the ectopic reporter activity in CCs detected when Su(H) binding to the *Him* enhancer was disrupted. We found that RNAi-mediated individual knockdowns of *gro*, *H*, or *CtBP* targeted to the cardiac mesoderm exhibits ectopic reporter activity by the *Him*^*WT*^ enhancer in CCs similar that observed in the case of the *Him*^*Su(H)*^ enhancer or the *Su(H)* RNAi (Fig 3E–3G´´´; S1 Table), consistent with our hypothesis of Su(H) acting as part of a transcriptional repressor complex with Gro, H, and CtBP in the regulation of *Him*.

### Su(H) activates transcription mediated by the *zfh1* enhancer in most pericardial cells

In contrast, we find that mutation of both Su(H) binding sites in the *zfh1* enhancer (*zfh1*^*Su(H)*^) (Fig 2; sequence in S4 File) abolishes reporter activity in most PCs (Fig 4A–4B´´´; S1 Table). A more detailed analysis with PCs labeled with relevant antibodies shows that *zfh1*^*Su(H)*^ eliminates reporter activity in the Odd-PCs and Tin-PCs but not in the Eve-PCs (Fig 5A–5B´´´, 5E–5F´´´, S1 Table). However, unlike in the case with *Him*, mutation of Su(H) sites in the *zfh1* enhancer does not result in any ectopic reporter activity in CCs. Knocking down the co-repressors Gro, H, or CtBP also fail to produce any ectopic reporter activity from the *zfh1* enhancer; the reporter is expressed in the same pattern as in an otherwise wild-type embryo (Fig 4C–4E´´´; S1 Table). Consistent with these observations, the same phenotype that was detected with the mutated *zfh1*^*Su(H)*^ enhancer—absence of reporter activity in CCs, Odd-PCs and Tin-PCs, but continued reporter activity in Eve-PCs—is also detected from the wild-type *zfh1* enhancer (*zfh1*^*WT*^) when Su(H) levels in the cardiac mesoderm are depleted by RNAi (Fig 5C–5D´´´, 5G–5H´´´; S1 Table). Thus, the *zfh1* enhancer requires Su(H) binding for activation in the majority of PCs, indicating that Su(H) functions as an activator of gene activity in this cardiac regulatory element, in contrast to its role in the *Him* enhancer where it serves as a repressor in CCs.

### Constitutive Notch signaling activates ectopic transcription from both the *Him* and *zfh1* enhancers in cardial cells

**Fig 6:**
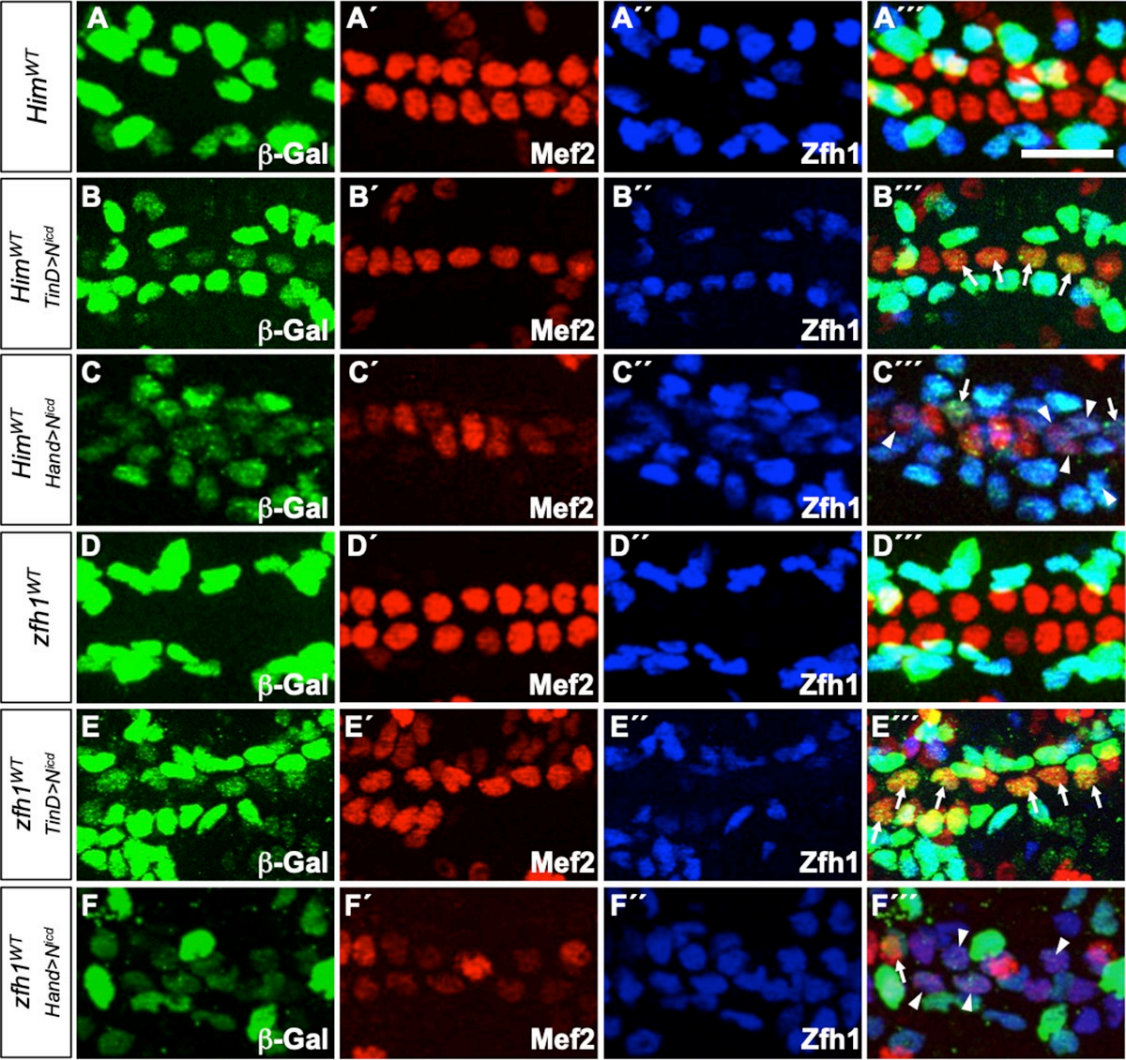
Constitutive Notch signaling activates ectopic transcription from both the *Him* and *zfh1* enhancers in CCs. (A-F´´´) *lacZ* reporter gene activity (β–galactosidase, green) driven by wild-type *Him* and *zfh1* enhancers in appropriate genotypes of stage 16 embryos. All CCs express Mef2 (red) while PCs are marked by Zfh1 (blue). Scale bar: 10 μm. (A-A´´´) The wild-type *Him* enhancer (*Him*^*WT*^) is active only in the Zfh1-expressing PCs. (B-B´´´) Overexpression of N^icd^ in all cells of the cardiac mesoderm by the early *TinD-GAL4* driver leads to the Mef2-expressing CCs exhibiting ectopic *Him*^*WT*^ enhancer-driven β–galactosidase reporter activity (arrows). The reduced number of CCs is a known consequence of the inhibition of cardiac progenitor specification by Notch signaling at an earlier phase of development [41–43]. (C-C´´´) Overexpression of N^icd^ in all cells of the heart by the later *Hand-GAL4* driver leads to some weakly Mef2-expressing CCs expressing both ectopic *Him*^*WT*^ enhancer-driven β–galactosidase reporter activity and ectopic Zfh1 protein (arrowheads) while other weakly Mef2-expressing CCs exhibit just *Him*^*WT*^ enhancer-driven reporter activity (arrows). (D-D´´´) The wild-type *zfh1* enhancer (*zfh1*^*WT*^) is also active only in the Zfh1-expressing PCs. (E-E´´´) Overexpression of N^icd^ in all cells of the cardiac mesoderm by the early *TinD-GAL4* driver leads to the Mef2-expressing CCs exhibiting ectopic *zfh1*^*WT*^ enhancer-driven β–galactosidase reporter activity (arrows). As in (B-B´´´), the inhibition of cardiac progenitor specification by ectopic Notch signaling results in a reduced number of CCs. (E-E´´´) Overexpression of N^icd^ in all cells of the heart by the later *Hand-GAL4* driver also leads to both ectopic *zfh1*^*WT*^ enhancer-driven reporter activity and ectopic Zfh1 protein in the weakly Mef2-expressing CCs (arrowheads).

The contrasting effects of eliminating Su(H) binding to the *Him* and *zfh1* enhancers raised the question of whether both these enhancers are regulated by the Notch signaling pathway in the CCs, Tin-PCs, and Odd-PCs. The processed intracellular domain of Notch (N^icd^) has previously been shown to associate with Su(H) to form a complex that both outcompetes the Su(H)-co-repressor complex for binding sites in the enhancers of target genes, thereby alleviating repression, and functions additionally as an activator complex [33–40]. We reasoned that ectopic expression of *N*^*icd*^ in all heart cells would result in the activation of wild-type *Him* and *zfh1* enhancers in CCs, where they are normally repressed or inactive [1–4], if and only if these enhancers were also responsive to Notch signaling. Ectopic expression of *N*^*icd*^ throughout the entire cardiac mesoderm and heart by the developmentally earlier *TinD-GAL4* driver [27] and the later *Hand-Gal4* driver [28] does indeed induce ectopic reporter activity in Mef2-expressing CCs from both *Him*^*WT*^ and *zfh1*^*WT*^ enhancers (Fig 6A–6F´´´; S4 Fig; S1 Table), thereby indicating that both Notch signaling and Su(H) are required for mediating the cell fate decision between Tin- and Odd-PCs and CCs. Of note, the level of reporter activity from both *Him*^*WT*^ and *zfh1*^*WT*^ enhancers in Mef2-expressing CCs appears higher in *Tin-D>N*^*icd*^ embryos than in *Hand>N*^*icd*^ embryos. Additionally, the level of reporter activity in PCs from both enhancers in *Tin-D>N*^*icd*^ embryos is similar to that in otherwise wild-type embryos. In contrast, the level of reporter activity from both enhancers in Zfh1- but not Mef2-expressing PCs in *Hand>N*^*icd*^ embryos is less than in PCs of otherwise wild-type embryos. However, the expression of Mef2 in CCs is also very weak in *Hand>N*^*icd*^ embryos, and ectopic Zfh1 protein is detected in many CCs, indicating that these CCs are in the process of partially adopting pericardial fates [41–43].

### Transcription mediated by the *zfh1* enhancer is dependent on Notch signaling in Odd-PCs and Tin-PCs, but not in Eve-PCs

The observation that eliminating Su(H) binding to the *zfh1* enhancer, either by mutating Su(H) binding sites or depleting the Su(H) TF itself, abrogated reporter activity in Odd-PCs and Tin-PCs, but not in Eve-PCs, demonstrated that the activation of this enhancer in Eve-PCs occurred independently of Su(H). Thus, a particularly germane question was whether the activation of the *zfh1* enhancer in Eve-PCs was also independent of Notch signaling, or whether Notch signaling utilized a TF other than Su(H) to activate *zfh1* transcription in Eve-PCs. To determine which of these hypotheses was correct, we depleted Notch in the entire heart by RNAi knockdown with the *TinD-GAL4* driver and examined reporter activity from the wild-type *zfh1* enhancer. If *zfh1*^*WT*^ reporter activity was eliminated as a result in all three PC subtypes, it would indicate that Notch signaling was also required for *zfh1* enhancer-mediated transcription in Eve-PCs. If instead, reporter activity was eliminated only in the Odd-PCs and Tin-PCs, but left unaffected in Eve-PCs, it would indicate that *zfh1* enhancer-mediated transcription in Eve-PCs is activated independently of Notch signaling. Our results show that *zfh1*^*WT*^ reporter activity is abolished in all PCs other than Eve-PCs when Notch is depleted (Fig 5I–5J´´´; S1 Table), thus demonstrating that in Eve-PCs, unlike in Odd-PCs and Tin-PCs, *zfh1* transcription is activated by a Notch-independent mechanism. Furthermore, the observation that the effect of the *Notch* knockdown on the *zfh1* enhancer completely phenocopies the effects of either mutating the Su(H) binding sites on the enhancer (Fig 5F–5F´´´) or knocking down *Su(H)* itself (Fig 5G–5H´´´) suggests that Notch and Su(H) function in the very same regulatory mechanism, one responsible for driving *zfh1* expression in Odd-PCs and Tin-PCs.

## Discussion

In both *Drosophila* and vertebrates, heart development entails the division, diversification, and differentiation of a pool of cardiac mesodermal cells into distinct cardiac subtypes [5, 44–47]. In *Drosophila*, the two major heart subtypes are cardial and pericardial cells. While each subtype can be divided even further based on gene expression, function, and morphology, all pericardial cells are defined by the exclusive PC-specific expression of both *Him* and *zfh1* at a transcriptional level [3, 4, 48, 49]. All cardial cells, in contrast, express *Mef2*, which is required for specifying the contractile myogenic nature of that subtype [29, 50–53].

Collectively, our results here demonstrate three distinct mechanisms by which these PC-specific genes have their transcription restricted to pericardial cells and are prevented from being expressed in cardial cells: one involving Su(H), an integral component of the Notch signaling pathway, repressing *Him* transcription in CCs; a second featuring Su(H) activating *zfh1* transcription in Odd-PCs and Tin-PCs; and a third in which *zfh1* transcription is activated in the Eve-PCs in a Su(H)-independent and Notch-independent manner. Furthermore, ectopic expression of *N*^*icd*^ in the cardiac mesoderm or in both CCs and PCs results in ectopic reporter activity from both the *Him* and *zfh1* enhancers in CCs, demonstrating that Notch signaling activates transcription mediated by both these enhancers. This conclusion is also illustrated by the ectopic expression of both the endogenous *Him* transcript and Zfh1 protein in CCs when *N*^*icd*^ is artificially expressed in all heart cells by the *Hand-GAL4* driver [3; this study]. Note that this ectopic expression of *N*^*icd*^ also reduces *Mef2* expression in CCs, suggesting that in a wild-type embryo, Notch signaling may also serve to inhibit *Mef2* expression, and thus also the CC-specific myogenic gene program that *Mef2* activates, in PCs. Additionally, since *Him* has previously been shown to act as a direct antagonist of *Mef2* function [54], the transcriptional activation of *Him* in PCs likely serves as yet another mechanism for repressing the CC-specific myogenic gene program in this subtype.

### Two different Notch signaling mechanisms regulate the expression of different PC-specific genes

Su(H) has previously been shown to associate with co-repressors in the absence of Notch signaling to form a repressor complex that binds dynamically and rapidly on and off the enhancers of target genes to prevent their transcription [33–40]. Notch signaling, initiated by ligand binding to Notch receptors, results in a proteolytic cleavage that releases N^icd^ from the cell membrane. N^icd^ then enters the nucleus and associates with Su(H) to form a N^icd^-Su(H) activator complex that competes dynamically with the Su(H)-co-repressor complex for Su(H) binding motifs on the enhancers. However, N^icd^ both enhances the recruitment of the N^icd^-Su(H) activator complex and its residence time at the enhancer, thereby favoring its presence over the Su(H)-co-repressor complex. This signaling pathway allows the activation of target genes to occur by one of two mechanisms: (i) a Notch-permissive process in which alleviating repression through the competitive displacement of the Su(H)-co-repressor complex is sufficient to initiate transcription of certain target genes due to the presence of other local TF activators that are then free to exert their positive effects, or (ii) a Notch-instructive process, which necessitates the inductive activating effect of the N^icd^-Su(H) complex for transcription initiation to occur [36–40].

**Fig 7:**
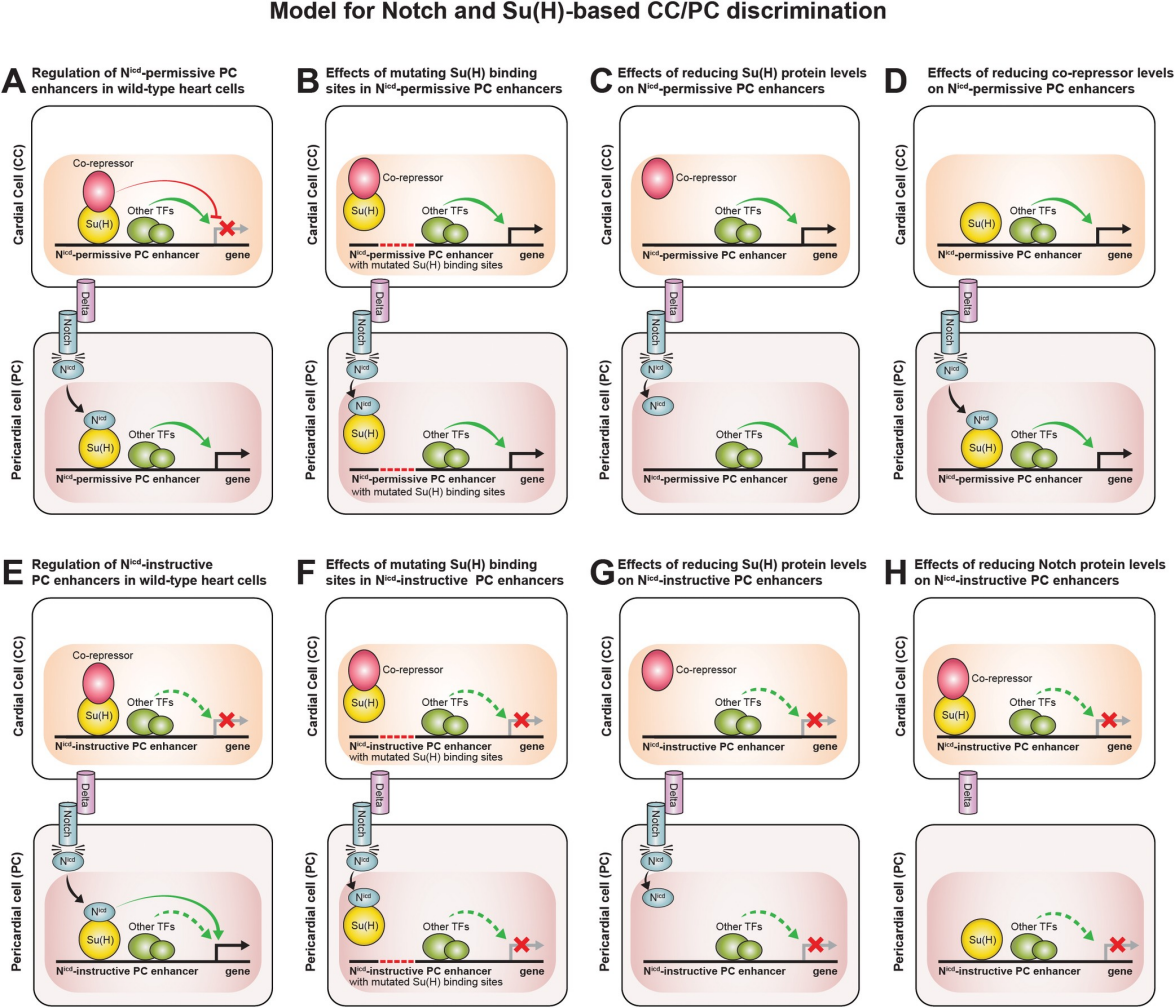
Schematic of Notch signaling pathway regulation of gene expression decisions between PCs and CCs via permissive and instructive mechanisms. Modes of regulation essential for the activation or repression of target genes are shown as solid green or red arrows, respectively, while those that are insufficient are shown as dashed arrows. (A, E) In cardial cells, PC genes such as *Him* with Notch-permissive PC enhancers are repressed by the Su(H)-co-repressor complex (A), while those with Notch-instructive PC enhancers like *zfh1* are prevented from being transcribed by the absence of the required N^icd^-Su(H) activator complex (E). The Delta ligand expressed by CCs activates the Notch receptor in neighboring PCs, with the resulting cleaved N^icd^ fragment associating with Su(H) to form a N^icd^-Su(H) complex that outcompetes the Su(H)-co-repressor complex for Su(H) binding sites in the enhancers. For the Notch-permissive PC enhancers, the consequent displacement of repressor complex binding in PCs is sufficient to initiate transcription due to the presence of other local TF activators (A); the Notch-instructive PC enhancers are necessarily activated by the N^icd^-Su(H) complex in PCs (E). (B, F) Mutating the Su(H) binding sites in either the Notch-permissive or the Notch-instructive PC enhancers prevents the Su(H)-co-repressor complex from binding to the enhancers in CCs and the N^icd^-Su(H) activator complex from binding in PCs. For Notch-permissive PC enhancers, the resulting alleviation of repression is sufficient to ectopically transcribe the associated gene in CCs (B); however, it is not adequate for the Notch-instructive PC enhancers which require the binding of the N^icd^-Su(H) activator complex to initiate transcription (F). The Su(H) site mutations in the Notch-instructive PC enhancers, however, also preclude the N^icd^-Su(H) activator complex from binding to the enhancer in PCs and thus prevent the target gene from being transcribed there (F); despite the inability of the N^icd^-Su(H) activator complex to bind, the absence of repression is sufficient to drive expression of target genes in PCs from the Notch-permissive PC enhancers (B). (C, G) Depleting the levels of the Su(H) TF prevents the Su(H)-co-repressor complex from forming and binding to the enhancers in CCs and the N^icd^-Su(H) activator complex from forming and binding to the enhancers in PCs. The absence of the repressor complex is sufficient to initiate ectopic transcription of the associated gene for Notch-permissive PC enhancers in CCs (C), while the absence of the N^icd^-Su(H) complex prevents transcription of the associated gene in both CCs and PCs for Notch-instructive PC enhancers (G). (D) Depleting the levels of a co-repressor prevents the Su(H)-co-repressor complex from forming and binding to the enhancers in CCs. The absence of the repressor complex thus initiates ectopic transcription of the associated gene for Notch-permissive PC enhancers in CCs. (H) Depleting the levels of Notch prevents the N^icd^-Su(H) activator complex from forming and binding to the enhancers in PCs. The absence of the activator complex thus prevents transcription of the associated gene for Notch-instructive PC enhancers in both CCs and PCs.

Our results indicate that the *Him* enhancer is a Notch-permissive enhancer, while the *zfh1* enhancer belongs to the class of Notch-instructive enhancers. In the absence of Notch signaling in CCs, co-repressors associate with Su(H) and the resulting repressor complex prevents transcription of PC-specific genes such as *Him* by a Notch-permissive mechanism (Fig 7A). In the case of PC-specific genes like *zfh1* that have Notch-instructive enhancers, it is the absence of the required N^icd^-Su(H) activator complex that prevents transcription initiation in CCs (Fig 7E). Since CCs express the ligand Delta [41], the Notch receptor is activated in neighboring PCs, with the subsequently cleaved N^icd^ fragment associating with Su(H) to form an activator complex that dynamically outcompetes the repressor complex in binding to the enhancers. For the Notch-permissive PC enhancers, the resulting displacement of the repressor complex by competition in PCs is sufficient to initiate transcription due to the presence of other local TF activators (Fig 7A); for the Notch-instructive PC enhancers, it is the inductive activating effect of the N^icd^-Su(H) complex in PCs which initiates transcription of PC genes (Fig 7E). These two distinct requirements for activation of transcription between Notch-permissive and Notch-instructive PC enhancers also explain the differences detected when Su(H) sites are mutated in the *Him* and *zfh1* enhancers, or when Su(H) levels are depleted. When the binding of the Su(H)-co-repressor complex to the *Him* Notch-permissive enhancer is prevented by either mutating the Su(H) motifs or knocking down Su(H) levels via cardiac mesoderm-targeted RNAi, reporter expression is de-repressed in CCs (Fig 7B and 7C). Similarly, knocking down a co-repressor prevents the repressor complex from being formed and binding to the *Him* Notch-permissive enhancer, resulting again in the de-repression of reporter activity in CCs (Fig 7D). In PCs, mere alleviation of repression by interfering with the binding of the Su(H)-co-repressor complex to the *Him* enhancer, whether via competition with the N^icd^-Su(H) complex in wild-type embryos, mutating the Su(H) binding sites, or knocking down Su(H) or a co-repressor in our experiments, is sufficient to ensure transcription (Fig 7A–7D).

However, since the *zfh1* PC enhancer is a Notch-instructive enhancer, elimination of the Su(H)-co-repressor complex is not sufficient to activate expression; in this case, transcriptional activation requires the binding of the N^icd^-Su(H) activator complex. Thus, when Su(H) binding is disrupted, the inability of the Su(H)-co-repressor complex to bind to the *zfh1* enhancer is not enough to bring about ectopic transcription in CCs (Fig 7F and 7G). Furthermore, the N^icd^-Su(H) activator complex can now also no longer bind to the enhancer in PCs, resulting in its inactivation in a subset of those cells (Fig 7F and 7G). Cardiac mesoderm-targeted knockdown of *Notch* also prevents the formation of the N^icd^-Su(H) activator complex and its binding to the *zfh1* enhancer in PCs, resulting in its similar inactivation in the same PC subset (Fig 7H). Our model suggests that transcription from both the *Him* Notch-permissive enhancer and the *zfh1* Notch-instructive enhancer will be initiated in CCs by ubiquitous Notch signaling in all cells of the heart, the former as a consequence of the alleviation of repression by the elimination of the Su(H)-co-repressor complex, and the latter due to direct activation by the N^icd^-Su(H) complex. This prediction is borne out by our experiments revealing that ectopic expression of both *Him* and *zfh1* occurs in CCs when the N^icd^ is targeted to both CCs and PCs (Fig 6A–6F´´´).

While the de-repression of reporter activity from *Him* enhancers in CCs detected in the case of the knockdown of co-repressors, the knockdown of Su(H), the mutation of the Su(H) motif in the enhancer, or the ectopic expression of N^icd^ argues strongly in favor of a Notch-permissive mechanism regulating *Him*, our results do not preclude additional modes of regulation. Su(H) could, in addition to being involved in the Notch-permissive mechanism described above, also activate a separate repressor of the *Him* enhancer. The presence of such a Su(H)-activated repressor might explain why, even when the Notch-permissive mechanism is activated by the ectopic *Hand-GAL4*-driven expression of *N*^*icd*^, the reporter activity from the *Him*^*WT*^ enhancer in both CCs and PCs, while present, is lower than that in PCs of otherwise wild-type embryos (Fig 6C).

This reduced level of reporter activity (compared to that in PCs of otherwise wild-type embryos), despite the ectopic presence of reporter activity in CCs, is seen from both the *Him*^*WT*^ and the *zfh1*^*WT*^ enhancers in response to the pan-cardiac expression of *N*^*icd*^ driven by *Hand-GAL4*. Additionally, while the level of reporter activity from both *Him*^*WT*^ and *zfh1*^*WT*^ enhancers in Mef2-expressing CCs appears higher in *Tin-D>N*^*icd*^ embryos than in *Hand>N*^*icd*^ embryos, it is only in the *Hand>N*^*icd*^ embryos that CCs exhibit both the expression of the endogenous Zfh1 protein and a considerable reduction in Mef2 expression levels. Notch signaling is utilized for two tightly controlled steps in *Drosophila* cardiogenesis: initially to restrict the number of dorsal mesodermal cells adopting the cardiac mesoderm fate between 6 and 8 h into development, and subsequently to bring about the differentiation of a subset of these cardiac mesoderm cells into PCs between 8 and 10 h into development [41–43]. Our attempt to ectopically initiate Notch signaling by either the *TinD-GAL4* or *Hand-GAL4*-driven expression of *N*^*icd*^ is unlikely to perfectly recapitulate the tightly regulated levels and timing of the later phase of endogenous Notch signaling, and is thus likely to cause imperfect cell type transformations by failing to activate the precise complement of necessary TFs. The relatively lower levels of reporter activity from both enhancers compared to that in wild-type PCs in response to the *Hand-GAL4*-driven pan-cardiac expression of *N*^*icd*^ or the absence of ectopic Zfh1 protein in the CCs of *TinD-GAL4*-driven *N*^*icd*^ expression likely reflect missing or reduced levels of the additional TFs necessary for PC-specific expression. One promising candidate for such a missing or reduced TF could be the Ets domain TF Pointed (Pnt), which has previously been shown to promote the pericardial fate in differentiating cardiac mesoderm cells [55–57]. Yet other such potential TF candidates necessary for producing wild-type levels of PC gene expression include PAR-domain protein 1 (Pdp1), and the *Drosophila* orthologs (CG17385, CG10321, and CG44002) of Zinc finger and BTB domain containing 14 (ZBTB14)—binding site motifs for these TFs were identified as positively weighted sequence features in machine learning-mediated discrimination of PC enhancers and subsequently validated empirically [57]. In this context, it is worth noting that, based on protein binding microarray data, putative binding sites for both Pnt and Pdp1 are present on both the *Him* and *zfh1* PC enhancers (S1 and S2 Files) [58, 59].

Regardless, mutations that eliminate the function of either *Notch* or its ligand *Delta* result in the overproduction of CCs at the expense of PCs [41–43], while loss-of-function mutations in *numb*, a gene encoding an antagonist of Notch, leads to the production of additional PCs at the expense of CCs [7, 28, 60]. Notch signaling thus plays a crucial role in establishing distinct cardiac cell subtypes by activating the pericardial gene program. Superficially, the process by which this Notch-mediated PC-specific expression is achieved appears quite similar in the case of both *Him* and *zfh1*: both genes are expressed specifically in PCs, not in CCs, and both are associated with enhancers that are enriched for Su(H) binding sites and drive transcription in the pericardial cells. However, we show in this study that this very feature, the enrichment of Su(H) binding sites in enhancers of the PC-specific genes, is utilized by two strikingly distinct regulatory mechanisms, Notch-permissive in the case of *Him* and Notch-instructive in the case of *zfh1*, to achieve ultimately the same result: specific expression of both these genes in PCs. Our results emphasize how very similar developmental outcomes can arise from identical inputs despite using very different regulatory mechanisms, thereby underscoring the importance of examining in detail even those aspects of developmental phenomena that superficially appear to be the same.

### What enhancer-specific features determine permissive versus instructive regulation via Notch?

The results presented in this study also raise the question of what enhancer-specific features might discriminate between Notch-permissive and Notch-instructive regulatory mechanisms. Our model suggests that while TFs binding to the Notch-instructive PC enhancers are unable to activate transcription in the absence of the N^icd^-Su(H) activator complex (Fig 7E), Notch-permissive PC enhancers possess binding sites for TFs that are sufficient to activate transcription once the enhancers are no longer repressed (Fig 7A). What specifically are these TFs that are sufficient to drive pericardial expression from Notch-permissive PC enhancers? Our future studies will attempt to identify these TFs discriminating between Notch-permissive and Notch-instructive PC enhancers by examining and comparing additional enhancers from each category.

### Two different regulatory mechanisms activate expression mediated by the same PC enhancer in distinct PC subtypes

A common theme in development is the repeated use of the same gene in multiple biological contexts, thus requiring its expression in several distinct spatial and/or temporal domains. The prevailing paradigm is that the transcription of a gene in each of these multiple domains is mediated by one or more enhancers unique for expression in that domain [10, 61–63]. Recently, however, we and other investigators discovered exceptions to this modular paradigm: several instances of individual *cis*-regulatory regions referred to as pleiotropic enhancers that are sufficient to drive expression in multiple domains [64–67].

**Fig 8:**
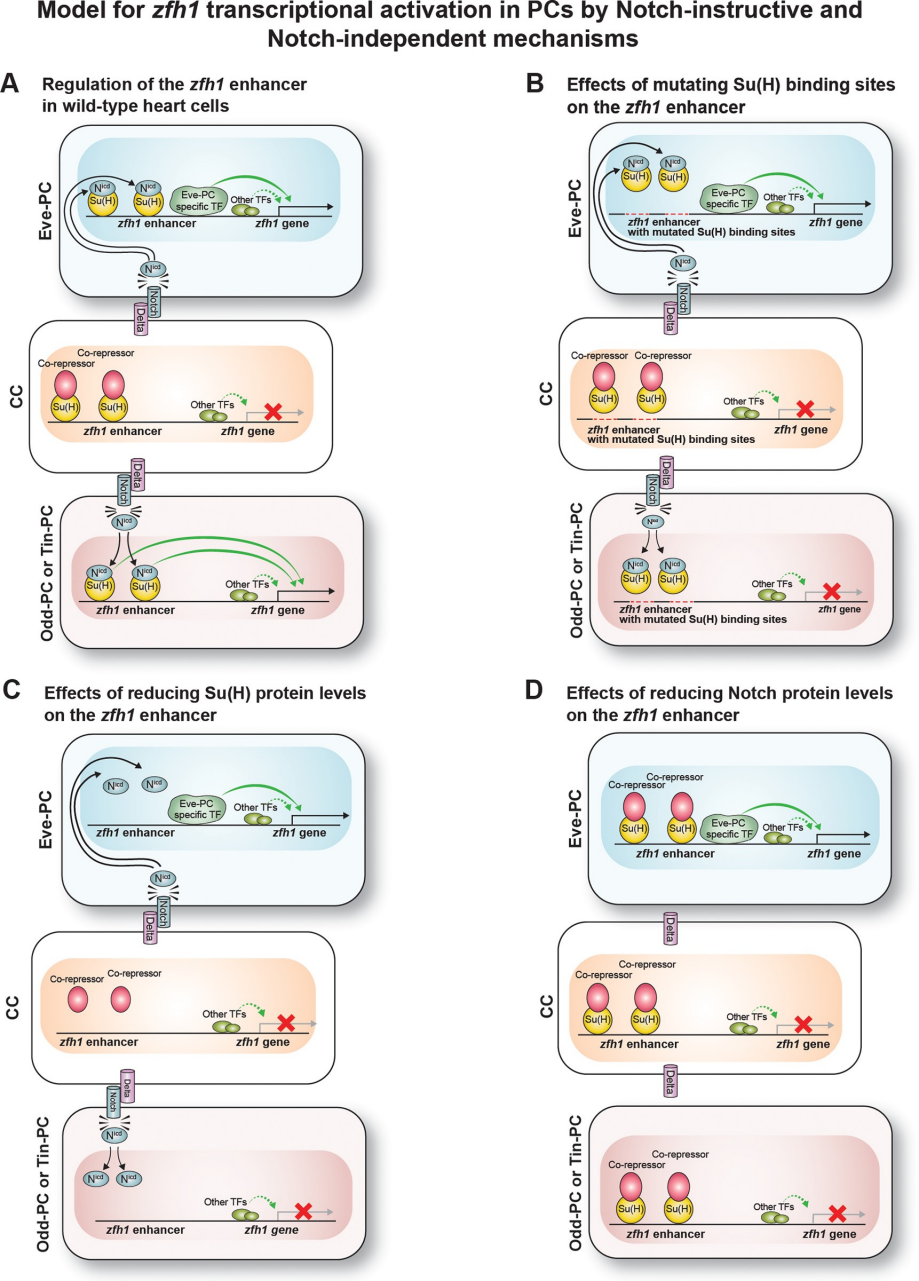
Model for the regulation of the *zfh1* enhancer by a Notch-instructive mechanism in CCs, Odd-PCs, and Tin-PCs, and by a Notch-independent mechanism in Eve-PCs. Modes of regulation essential for the transcriptional activation of *zfh1* are shown as solid green arrows, while those that are insufficient are shown as dashed arrows. (A) For wild-type embryos, the absence of both the N^icd^-Su(H) activator complex and the Eve-PC-specific TF binding to the *zfh1* enhancer in cardial cells prevents *zfh1* from being transcribed. The Delta ligand expressed by CCs activates Notch receptor in neighboring PCs, with the resulting cleaved N^icd^ fragment associating with Su(H) and outcompeting the co-repressor in Odd-PCs and Tin-PCs. In the Odd-PCs and Tin-PCs, the binding of the N^icd^-Su(H) activator complex to the *zfh1* enhancer initiates transcription. In the Eve-PCs, the binding of the Eve-PC-specific TF to the *zfh1* enhancer is sufficient to activate transcription. (B) Mutating the Su(H) binding sites prevents the N^icd^-Su(H) activator complex from binding to *zfh1* enhancer in all CCs and PCs. In Odd-PCs and Tin-PCs which also lack the Eve-specific TF, the absence of binding of either of these activating complexes/TFs prevents *zfh1* transcription. In contrast, in Eve-PCs, the Eve-PC-specific TF is present, binds to the *zfh1* enhancer, and is sufficient to activate transcription. (C) Depleting the levels of the Su(H) TF prevents the N^icd^-Su(H) activator complex from forming and binding to the enhancers in all PCs. In Odd-PCs and Tin-PCs, its absence and a lack of the Eve-PC-specific TF prevents *zfh1* transcription. In Eve-PCs, the binding of the Eve-PC-specific TF to the *zfh1* enhancer is again sufficient to initiate transcription. (D) Depleting the levels of Notch protein also prevents the N^icd^-Su(H) activator complex from forming and binding to the enhancers in all PCs. As in (C), the absence of both the N^icd^-Su(H) complex and the Eve-PC-specific TF prevents *zfh1* transcription in Odd-PCs and Tin-PCs. But in Eve-PCs, where the Eve-PC-specific TF is present and able to bind to the *zfh1* enhancer, transcription is activated.

Here we show that the *zfh1* enhancer constitutes yet another rare example of such a pleiotropic enhancer, being able to activate transcription in Odd-PCs, Tin-PCs, and Eve-PCs. What is particularly striking about the *zfh1* enhancer, however, is that it activates expression in Odd-PCs and Tin-PCs by a Notch-instructive mechanism, and in the Eve-PCs by a Notch-independent mechanism. Our model attempting to explain this phenomenon is presented in Fig 8. We propose that the *zfh1* enhancer contains one or more binding site(s) for a TF expressed exclusively in Eve-PCs in addition to the two Su(H) binding sites, and that the binding of this Eve-PC-specific TF to the enhancer is sufficient to initiate transcription (Fig 8A). Thus, when the binding of the N^icd^-Su(H) activator complex to the Notch-instructive *zfh1* enhancer is prevented by mutating the Su(H) motifs (Fig 8B), using RNAi to deplete Su(H) (Fig 8C), or using RNAi to deplete Notch (Fig 8D), binding of the Eve-PC-specific TF to the enhancer is still sufficient to activate transcription in the Eve-PCs. However, in Odd-PCs and Tin-PCs, where the Eve-PC-specific TF is not expressed, the absence of binding of both the N^icd^-Su(H) complex and the Eve-PC-specific TF prevents transcription from being activated (Fig 8B–8D). Note that our model does not necessarily preclude *zfh1* transcription in Eve-PCs from being activated by a Notch-instructive mechanism in addition to the Notch-independent mechanism, thereby potentially providing this process with a degree of redundancy; however, we are unable to functionally assess whether this potential redundancy exists without knowing the identity of the Eve-PC-specific TF.

### What is the TF responsible for Notch-independent *zfh1* expression in Eve-PCs?

An obvious candidate for the TF responsible for the Notch-independent expression of *zfh1* in Eve-PCs is Eve itself since it is expressed exclusively in the Eve-PCs of the heart. Furthermore, in anterior corner cell (aCC) motor neurons, Eve transcriptionally activates the GATA TF Grain (Grn), and both Eve and Grn activate *zfh1* expression [68]. Finally, based on protein binding microarray data, putative binding sites for both Eve and Grn are present on the *zfh1* PC enhancer sequence (S2 File) [58, 59, 69]. However, since we were unsuccessful in completely eliminating Eve in Eve-PCs via RNAi knockdown as determined by immunohistochemical staining, we are unable to assess at present if Eve is indeed the TF driving the Eve-PC-specific Notch-independent *zfh1* expression. We intend to address the identity of this Eve-PC-specific TF in future studies by clonal mosaic-based elimination of Eve in specific cells, and by combining single cell transcriptomic data with enhancer dissection.

Collectively, our study shows that three distinct regulatory mechanisms, Notch-permissive, Notch-instructive, and Notch-independent, are responsible for the exclusive PC-specific expression of at least two genes that confer pericardial identity, and thus for cardiac subtype specification. The use of multiple mechanisms for achieving ultimately the same goal likely reflects both the complexity of cardiac subtype specification and the degree of redundancy necessary for increased robustness in the development of critical organs such as the heart.

## Supporting information

S1 Fig Diagram of the *pWattB-nlacZ* vector and its Multiple Cloning Site (MCS). Relevant features of the vector and usable restriction sites in the MCS are shown.
(TIF)

S2 Fig Controls for RNAi assays with the *Him* enhancer. (A-F´´´) *lacZ* reporter gene activity (β–galactosidase, green) driven by the *Him*^*WT*^ enhancers in appropriate genotypes of stage 16 embryos. All CCs express Mef2 (red) while PCs are marked by Zfh1 (blue). Scale bar: 10 μm. Representative images of *Him*^*WT*^–driven reporter activity in embryos containing one copy of the *TinD-GAL4* driver (A-A´´´), one copy of the UAS-RNAi construct *Su(H)*^*HMS05748*^ (B-B´´´), one copy of the UAS-RNAi construct *Su(H)*^*HM05110*^ (C-C´´´), one copy of the UAS-RNAi construct *gro*^*KK108953*^ (D-D´´´), one copy of the UAS-RNAi construct *H*^*GD1458*^ (E-E´´´), and one copy of the UAS-RNAi construct *CtBP*^*KK108401*^ (F-F´´´). Note that the reporter activity is similar to that from *Him*^*WT*^ enhancers in otherwise wild-type embryos.
(TIF)

S3 Fig Controls for RNAi assays with the *zfh1* enhancer. (A-H´´´) *lacZ* reporter gene activity (β–galactosidase, green) driven by the *zfh1*^*WT*^ enhancers in appropriate genotypes of stage 16 embryos. All CCs express Mef2 (red) while PCs are marked by Zfh1 (blue). Scale bar: 10 μm. Representative images of *zfh1*^*WT*^–driven reporter activity in embryos containing one copy of the *TinD-GAL4* driver (A-A´´´), one copy of the UAS-RNAi construct *Su(H)*^*HMS05748*^ (B-B´´´), one copy of the UAS-RNAi construct *Su(H)*^*HM05110*^ (C-C´´´), one copy of the UAS-RNAi construct *gro*^*KK108953*^ (D-D´´´), one copy of the UAS-RNAi construct *H*^*GD1458*^ (E-E´´´), one copy of the UAS-RNAi construct *CtBP*^*KK108401*^ (F-F´´´), one copy of the UAS-RNAi construct *N*^*HMS00001*^ (G-G´´´), and one copy of the UAS-RNAi construct *N*^*HMS00009*^ (H-H´´´). Note that the reporter activity is similar to that from *zfh1*^*WT*^ enhancers in otherwise wild-type embryos.
(TIF)

S4 Fig *TinD-GAL4* and *Hand-GAL4* drive expression in all heart cells. (A-B´´´) *UAS*-*lacZ* reporter gene activity (β–galactosidase, green) driven by *TinD-GAL4* (A) and *Hand-GAL4* (B) drivers in stage 16 embryos. All CCs express Mef2 (red) while PCs are marked by Zfh1 (blue). Note that all CCs and PCs express the reporter, albeit at somewhat different levels.
(TIF)

S1 Table Reporter activity from the *Him* and *zfh1* enhancers in different genotypes. Genotypes of the embryos used to examine activity from transgenic reporter constructs via fluorescent immunohistochemistry, the number of embryos examined for each genotype as well as the number of embryos exhibiting specific phenotypes, and a list of the figures showing representative embryos of each genotype.
(XLSX)

S1 File Sequence of the wild-type *Him* enhancer. The Su(H) binding site is underscored and highlighted in yellow. Putative Pnt and Pdp1 binding sites based on protein binding microarray data are underscored and highlighted in green and cyan, respectively.
(PDF)

S2 File Sequence of the wild-type *zfh1* enhancer. The two Su(H) binding sites are underscored and highlighted in yellow. Putative Pnt, Pdp1, Eve and Grn binding sites based on protein binding microarray data are underscored and highlighted in green, cyan, magenta, and grey, respectively.
(PDF)

S3 File Sequence of the mutated *Him* enhancer. The mutated Su(H) binding site is underscored and highlighted in yellow. The single nucleotide substitution used to create a binding site mutation that eliminates Su(H) binding is shown in red lowercase.
(PDF)

S4 File Sequence of the mutated *zfh1* enhancer. The mutated Su(H) binding sites are underscored and highlighted in yellow. The single nucleotide substitutions used to create binding site mutations that eliminate Su(H) binding are shown in red lowercase.
(PDF)

S5 File Assessment of RNAi knockdown efficacy with reverse transcription quantitative real-time PCR (RT-qPCR). The methodology, primers, and results of the RT-qPCR analysis are provided.
(PDF)

S6 File Sequence of the *pWattB-nlacZ* cloning vector. The complete sequence of this cloning vector, also available from GenBank with the accession number MT747949, is provided in GenBank flat file format.
(PDF)
