# Supplementary figures and images for: Three distinct mechanisms, Notch instructive, permissive, and independent, regulate the expression of two different pericardial genes to specify cardiac cell subtypes
Source: PLoS One. 2020 Oct 27;15(10):e0241191. doi: 10.1371/journal.pone.0241191 (PMC7591092; doi:10.1371/journal.pone.0241191)

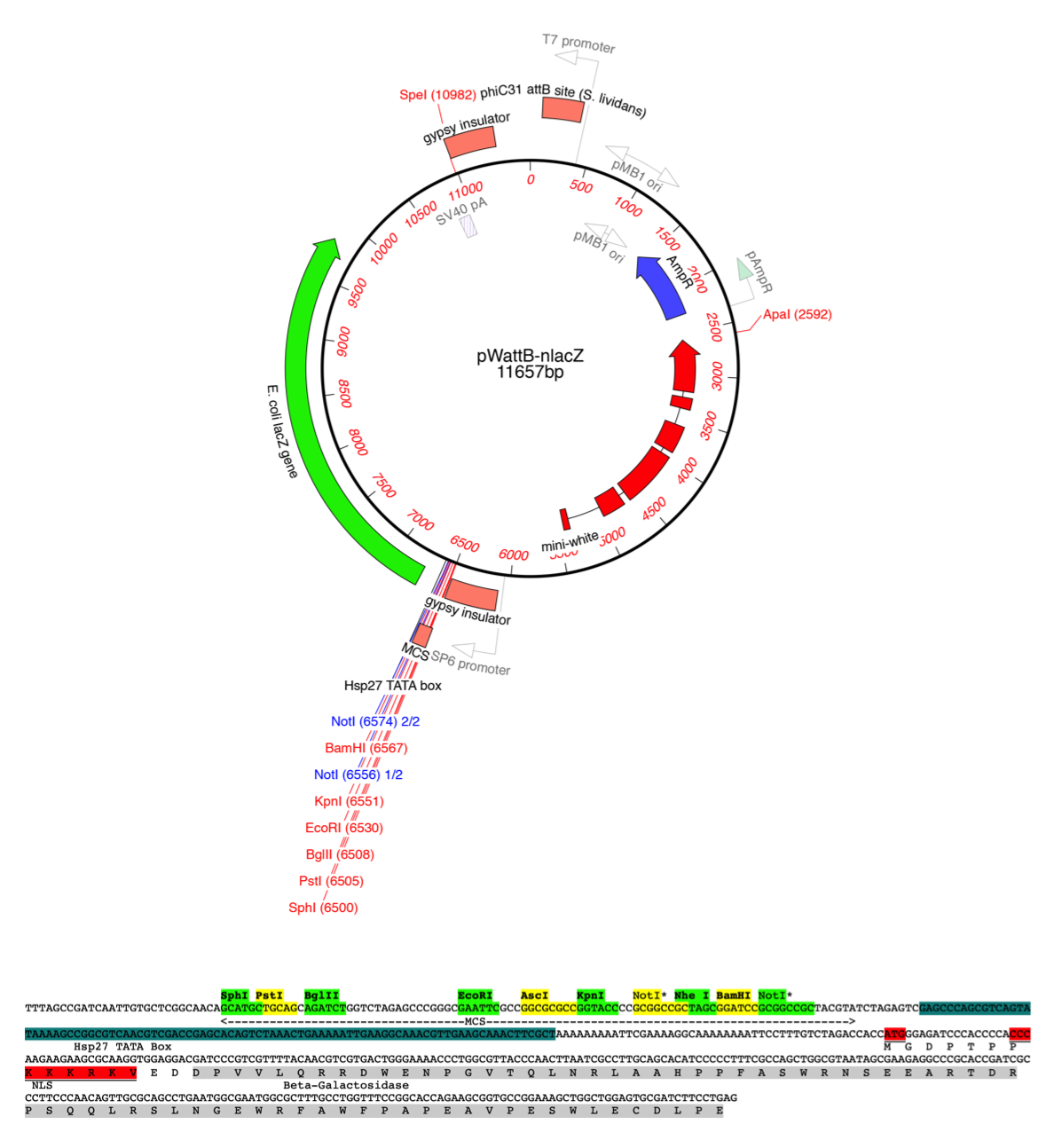

Supplement: S1 Fig — Relevant features of the vector and usable restriction sites in the MCS are shown. (TIF) [file pone.0241191.s001.tif]

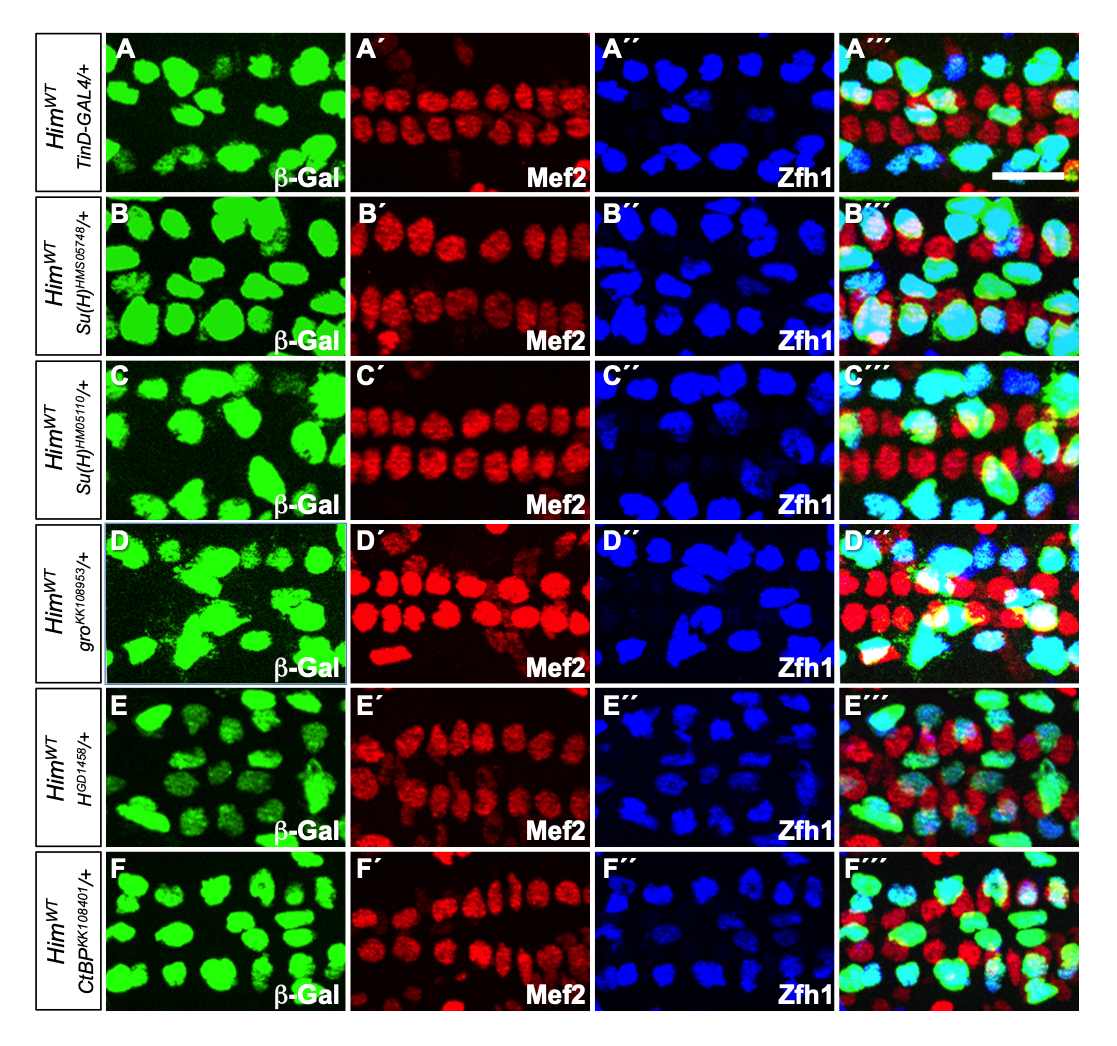

Supplement: S2 Fig — (A-F´´´) lacZ reporter gene activity (β–galactosidase, green) driven by the HimWT enhancers in appropriate genotypes of stage 16 embryos. All CCs express Mef2 (red) while PCs are marked by Zfh1 (blue). Scale bar: 10 μm. Representative images of HimWT–driven reporter activity in embryos containing one copy of the TinD-GAL4 driver (A-A´´´), one copy of the UAS-RNAi construct Su(H)HMS05748 (B-B´´´), one copy of the UAS-RNAi construct Su(H)HM05110 (C-C´´´), one copy of the UAS-RNAi construct groKK108953 (D-D´´´), one copy of the UAS-RNAi construct HGD1458 (E-E´´´), and one copy of the UAS-RNAi construct CtBPKK108401 (F-F´´´). Note that the reporter activity is similar to that from HimWT enhancers in otherwise wild-type embryos. (TIF) [file pone.0241191.s002.tif]

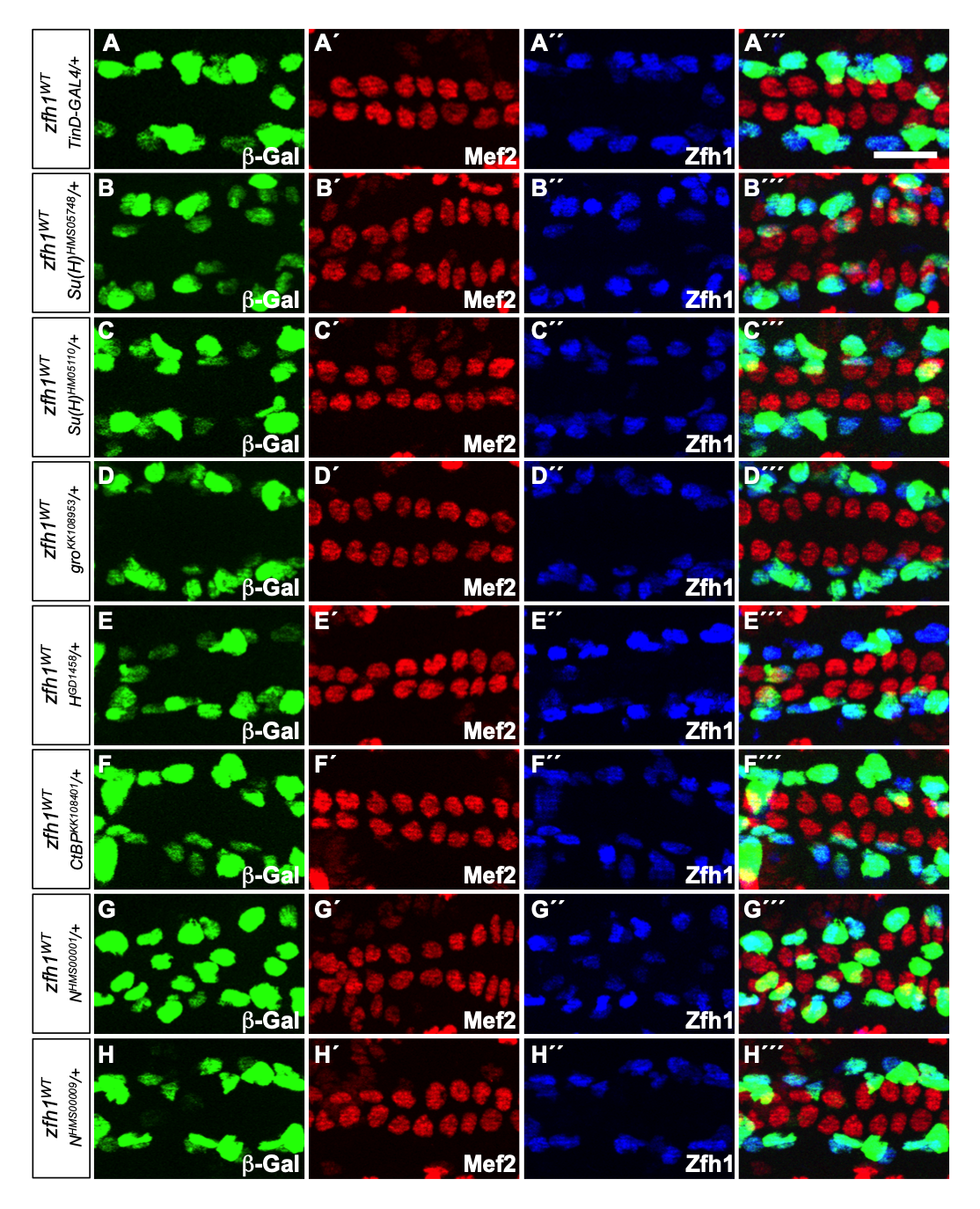

Supplement: S3 Fig — (A-H´´´) lacZ reporter gene activity (β–galactosidase, green) driven by the zfh1WT enhancers in appropriate genotypes of stage 16 embryos. All CCs express Mef2 (red) while PCs are marked by Zfh1 (blue). Scale bar: 10 μm. Representative images of zfh1WT–driven reporter activity in embryos containing one copy of the TinD-GAL4 driver (A-A´´´), one copy of the UAS-RNAi construct Su(H)HMS05748 (B-B´´´), one copy of the UAS-RNAi construct Su(H)HM05110 (C-C´´´), one copy of the UAS-RNAi construct groKK108953 (D-D´´´), one copy of the UAS-RNAi construct HGD1458 (E-E´´´), one copy of the UAS-RNAi construct CtBPKK108401 (F-F´´´), one copy of the UAS-RNAi construct NHMS00001 (G-G´´´), and one copy of the UAS-RNAi construct NHMS00009 (H-H´´´). Note that the reporter activity is similar to that from zfh1WT enhancers in otherwise wild-type embryos. (TIF) [file pone.0241191.s003.tif]

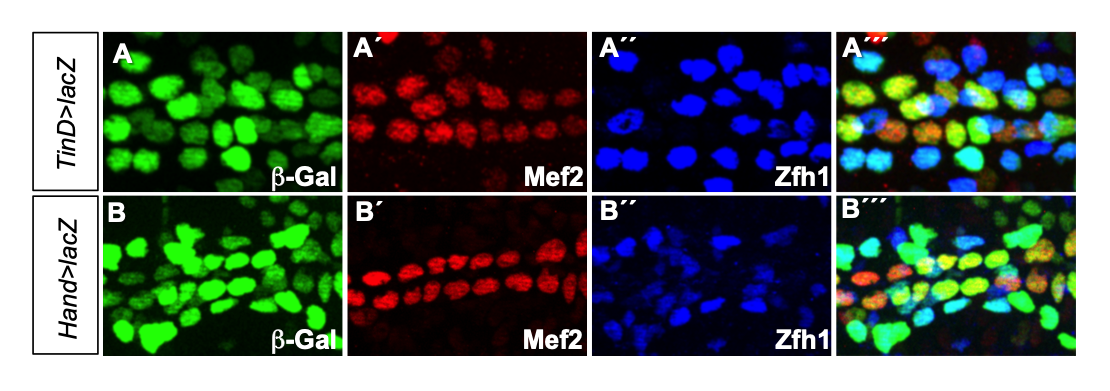

Supplement: S4 Fig — (A-B´´´) UAS-lacZ reporter gene activity (β–galactosidase, green) driven by TinD-GAL4 (A) and Hand-GAL4 (B) drivers in stage 16 embryos. All CCs express Mef2 (red) while PCs are marked by Zfh1 (blue). Note that all CCs and PCs express the reporter, albeit at somewhat different levels. (TIF) [file pone.0241191.s004.tif]
