# Supplementary material for: Three distinct mechanisms, Notch instructive, permissive, and independent, regulate the expression of two different pericardial genes to specify cardiac cell subtypes
Source: PLoS One. 2020 Oct 27;15(10):e0241191. doi: 10.1371/journal.pone.0241191 (PMC7591092; doi:10.1371/journal.pone.0241191)
Supplement: S1 File — The Su(H) binding site is underscored and highlighted in yellow. Putative Pnt and Pdp1 binding sites based on protein binding microarray data are underscored and highlighted in green and cyan, respectively. (PDF) [file pone.0241191.s006.pdf]

Him^{WT} enhancer sequence

```
        10        20        30        40        50        60
TGGCATCAGTGGGTCACAGGGATTACGGCTGGCTGGGATTCCAGAGCCAGATCTTTTTCA

                                  Putative Pnt binding site
                                            |
        70        80        90       100    |  110       120
GCCAAAACTTTCAGCTTTCGAAGACCTCAAGCGATAGGAGAGTGTCGGAAGTCCAGAAAT

       130       140       150       160       170       180
AGACGCGTAGCACATAAATTATGGATCGTATCGAGTATCGATTAGCCCGGGACAAGCGAA

       190       200       210       220       230       240
GCGATAGGGAGACATATTTTTATTACCCTCTCGGGGACCTGCACTTGTTGGCTTCGCTTC

                                          Su(H) binding site
                                                       |
       250       260       270       280       290     | 300
TATGAAAGATCCCTCTACCATATCACGTATGTGGGCTCCCCCAATCGAACCGAGTTGTGG

       310       320       330       340       350       360
GAAATGTTTTCCCAGGCCAACAGCTAATTGTCACTCCAAGGGTTGTCCCCGCAGCCCAGA

       370       380       390       400       410       420
CGACAGATAAGCGGGCAAGTGAAGCCCAGCGATCTGAGTCAAGTGAAGGGCTTCAATTTC

                     Putative Pdp1 binding site
                                  |
       430       440       450    |  460       470       480
TTTCCCGAGTGGAACTGGGATATCGAAATTACATTTGTAACAGACGTTTTAGTCCGCAAT

   Putative Pdp1 binding site
                |
       490      |500       510       520       530       540
CCTCAGCTAATGGGACTTACGAACATATATTCATCTGAAATTCAAGAACATGCGCACTTA

       550       560       570       580       590
AAGAGCAGGGAAGTCGCACACGCGCAAGTCAGGCGCTCAAAAAGGGATCTTCGGA
```
