# Supplementary material for: Three distinct mechanisms, Notch instructive, permissive, and independent, regulate the expression of two different pericardial genes to specify cardiac cell subtypes
Source: PLoS One. 2020 Oct 27;15(10):e0241191. doi: 10.1371/journal.pone.0241191 (PMC7591092; doi:10.1371/journal.pone.0241191)
Supplement: S2 File — The two Su(H) binding sites are underscored and highlighted in yellow. Putative Pnt, Pdp1, Eve and Grn binding sites based on protein binding microarray data are underscored and highlighted in green, cyan, magenta, and grey, respectively. (PDF) [file pone.0241191.s007.pdf]

zfh1^{WT} enhancer sequence

```
                   Su(H) binding site
                            |
         10        20        30        40        50        60
ACGAGCACTTTTCACGAGGGGGAAAATCTTCCCACACGAGAACCACACACTTGACTAAAA

         70        80        90       100       110       120
CCACCGCCCCAATCAAGTTCTAACCCTTGAAAAATAGAAGGGGGAAAATGGGGCTCAGAC

              Putative Pnt binding site
                          |
        130       140     |150       160       170       180
CGCTGTACATGCATTTTGGGGACGATGAGGAAATTTGACATTTCCCCGCCGCCACTTAAA

Putative Pdp1 binding site
  |
  |     190       200       210       220       230       240
TGTCACATAAAGTGTTAAACGAAATGAGGCAAAGGCGGCCGCAGAACAGAGGACACTTGA

        250       260       270       280       290       300
GATTTCGATTCCGATGCCGAGAGTCCGATGTTTGTGGCGACCCCAAAAATGTGCATCACT

        310       320       330       340       350       360
CTGCACCACCGAGGCCAGCACCAGCAGCACCACCACCACCACCACCACACCACACCACTT

                                Putative Eve binding site
                                            |
        370       380       390       400   |  410       420
TCTATGCCATATAGAACCACCACATTCTGAAAAGTATTTACGCTGTCATTACTTTTCGGT

        430       440       450       460       470       480
TCGGTTTCGAATTGAATTGGTTTCGGGTTCTTTTGTTTTTCGGGGCCGCGCAAAGTTTAC

                                          Su(H) binding site
                                                         |
Putative Eve binding site                                |
  |                                                      |
  |     490       500       510       520       530       540
TGCTAATTAAAAATTGTGGAAAAACGAGAAACATGCATGCAATTCAATCCCAAGGAGTGT

                    Putative Pdp1 binding site
                                 |
      Putative Grn binding site  |
                     |           |
        550       560|      570  |    580       590       600
GGGAATGCGATGACCCATCTGGAGATCTGAGATTTACAGCAAACACCCCGAAAAGGATTG
```

zfh1^{WT} enhancer sequence (continued)

```
                                                    Putative Eve binding site
                                                                            |
       610       620       630       640       650       660
CCTTTTCCCAGAGTCCTTTGCTTCATCTTCTCGTCGATCCGACGATTGTAAAGTGAAATT

                                                    Putative Pdp1 binding site
                                                                 |
     Putative Pdp1 binding site                                  |
                                   |                             |
       670       680       690       700       710       720
AATTGATTCCTCCGTCGATGACGTCAATGCGGAACCATCGAAAGTACTTAGCTAACTCTA

Putative Pdp1 binding site
  |
  |    730       740       750       760       770       780
TTTTATGTGGGTACCAGCAGCGGTGGGTGAGAATTTCCAAAGTCGTGGAAGCCCAAGCTA

                     Putative Pdp1 binding site
                                 |
       790       800       810   |   820       830       840
GTAGCAATCGGAAAATGATCGACAAATCCAATTTAAATAAAGAACCTACAGTGGGTGTTG

                                                     Putative Eve binding site
                                                                  |
       850       860       870       880       890       900
CCTTCGAGAGCCTGGGAAGAAAATCCTTGAGTGCGAATGCGGGACGTGGTAATTGAAGGT

       910       920       930
ACTCGCCATTGAACTCGGATGCATTTCGAT
```
