# Supplementary material for: Three distinct mechanisms, Notch instructive, permissive, and independent, regulate the expression of two different pericardial genes to specify cardiac cell subtypes
Source: PLoS One. 2020 Oct 27;15(10):e0241191. doi: 10.1371/journal.pone.0241191 (PMC7591092; doi:10.1371/journal.pone.0241191)
Supplement: S3 File — The mutated Su(H) binding site is underscored and highlighted in yellow. The single nucleotide substitution used to create a binding site mutation that eliminates Su(H) binding is shown in red lowercase. (PDF) [file pone.0241191.s008.pdf]

Him$^{Su(H)}$ enhancer

```
        10        20        30        40        50        60
TGGCATCAGTGGGTCACAGGGATTACGGCTGGCTGGGATTCCAGAGCCAGATCTTTTTCA

        70        80        90       100       110       120
GCCAAAACTTTCAGCTTTCGAAGACCTCAAGCGATAGGAGAGTGTCGGAAGTCCAGAAAT

       130       140       150       160       170       180
AGACGCGTAGCACATAAATTATGGATCGTATCGAGTATCGATTAGCCCGGGACAAGCGAA

       190       200       210       220       230       240
GCGATAGGGAGACATATTTTTATTACCCTCTCGGGGACCTGCACTTGTTGGCTTCGCTTC

        Su(H) binding site mutated from YGTGDGAA to YGTGDCAA
                                                       |
       250       260       270       280       290     | 300
TATGAAAGATCCCTCTACCATATCACGTATGTGGGCTCCCCCAATCGAACCGAGTTGTGG

       310       320       330       340       350       360
cAAATGTTTTCCCAGGCCAACAGCTAATTGTCACTCCAAGGGTTGTCCCCGCAGCCCAGA

       370       380       390       400       410       420
CGACAGATAAGCGGGCAAGTGAAGCCCAGCGATCTGAGTCAAGTGAAGGGCTTCAATTTC

       430       440       450       460       470       480
TTTCCCGAGTGGAACTGGGATATCGAAATTACATTTGTAACAGACGTTTTAGTCCGCAAT

       490       500       510       520       530       540
CCTCAGCTAATGGGACTTACGAACATATATTCATCTGAAATTCAAGAACATGCGCACTTA

       550       560       570       580       590
AAGAGCAGGGAAGTCGCACACGCGCAAGTCAGGCGCTCAAAAAGGGATCTTCGGA
```
