# Supplementary material for: Three distinct mechanisms, Notch instructive, permissive, and independent, regulate the expression of two different pericardial genes to specify cardiac cell subtypes
Source: PLoS One. 2020 Oct 27;15(10):e0241191. doi: 10.1371/journal.pone.0241191 (PMC7591092; doi:10.1371/journal.pone.0241191)
Supplement: S4 File — The mutated Su(H) binding sites are underscored and highlighted in yellow. The single nucleotide substitutions used to create binding site mutations that eliminate Su(H) binding are shown in red lowercase. (PDF) [file pone.0241191.s009.pdf]

zfh1$^{Su(H)}$ enhancer sequence

```
  Su(H) binding site mutated from YGTGDGAA to YGTGDCAA
                            |
        10        20        30        40        50        60
ACGAGCACTTTTCACGAGGGGGAAAATCTTgCCACACGAGAACCACACACTTGACTAAAA

        70        80        90       100       110       120
CCACCGCCCCAATCAAGTTCTAACCCTTGAAAAATAGAAGGGGGAAAATGGGGCTCAGAC

       130       140       150       160       170       180
CGCTGTACATGCATTTTGGGGACGATGAGGAAATTTGACATTTCCCCGCCGCCACTTAAA

       190       200       210       220       230       240
TGTCACATAAAGTGTTAAACGAAATGAGGCAAAGGCGGCCGCAGAACAGAGGACACTTGA

       250       260       270       280       290       300
GATTTCGATTCCGATGCCGAGAGTCCGATGTTTGTGGCGACCCCAAAAATGTGCATCACT

       310       320       330       340       350       360
CTGCACCACCGAGGCCAGCACCAGCAGCACCACCACCACCACCACCACACCACACCACTT

       370       380       390       400       410       420
TCTATGCCATATAGAACCACCACATTCTGAAAAGTATTTACGCTGTCATTACTTTTCGGT

       430       440       450       460       470       480
TCGGTTTCGAATTGAATTGGTTTCGGGTTCTTTTGTTTTTCGGGGCCGCGCAAAGTTTAC

       Su(H) binding site mutated from YGTGDGAA to YGTGDCAA
                                                         |
       490       500       510       520       530       540
TGCTAATTAAAAATTGTGGAAAAACGAGAAACATGCATGCAATTCAATCCCAAGGAGTGT

       550       560       570       580       590       600
GGcAATGCGATGACCCATCTGGAGATCTGAGATTTACAGCAAACACCCCGAAAAGGATTG

       610       620       630       640       650       660
CCTTTTCCCAGAGTCCTTTGCTTCATCTTCTCGTCGATCCGACGATTGTAAAGTGAAATT

       670       680       690       700       710       720
AATTGATTCCTCCGTCGATGACGTCAATGCGGAACCATCGAAAGTACTTAGCTAACTCTA

       730       740       750       760       770       780
TTTTATGTGGGTACCAGCAGCGGTGGGTGAGAATTTCCAAAGTCGTGGAAGCCCAAGCTA

       790       800       810       820       830       840
GTAGCAATCGGAAAATGATCGACAAATCCAATTTAAATAAAGAACCTACAGTGGGTGTTG

       850       860       870       880       890       900
CCTTCGAGAGCCTGGGAAGAAAATCCTTGAGTGCGAATGCGGGACGTGGTAATTGAAGGT

       910       920       930
ACTCGCCATTGAACTCGGATGCATTTCGAT
```
