# Supplementary material for: Three distinct mechanisms, Notch instructive, permissive, and independent, regulate the expression of two different pericardial genes to specify cardiac cell subtypes
Source: PLoS One. 2020 Oct 27;15(10):e0241191. doi: 10.1371/journal.pone.0241191 (PMC7591092; doi:10.1371/journal.pone.0241191)
Supplement: S5 File — The methodology, primers, and results of the RT-qPCR analysis are provided. (PDF) [file pone.0241191.s010.pdf]

Assessment of RNAi knockdown efficacy with reverse transcription quantitative real-time PCR (RT-qPCR)

Materials and Methods

Embryo preparation for total RNA extraction

Stage 13-16 embryos (9-15.5 h after egg laying) of control and appropriate RNAi knockdown genotypes were collected after being raised at 29°C. Control embryos contained one copy each of the *TinD-GAL4* driver and *UAS-Dcr-2*, while RNAi knockdown embryos possessed one copy each of the *TinD-GAL4* driver and *UAS-Dcr-2* in addition to one copy of the relevant UAS-RNAi construct (Table 1). Embryos were dechorionated by immersion in 50% bleach for 5 m followed by a quick rinse with 0.1% Triton-X and then with water.

Total RNA isolation and quantification

Total RNA was isolated immediately after the dechorionation and rinse step using the Direct Zol™ RNA MicroPrep Kit (Zymo Research) according to the manufacturer's recommendation which included in-column DNase I treatment to remove genomic DNA. Total RNA was eluted in 10 µl of RNase/DNase-Free water. One µl was used to quantify total RNA concentration and quality using a Thermo Scientific™ NanoDrop™ One Microvolume UV-Vis Spectrophotometer (Table 1).

Embryo	Genotype	Total RNA Concentration	260/280 Ratio	260/230 Ratio
Control	*TinD-GAL4 UAS-Dcr-2/+*	1.80 µg/µl	2.18	2.58
Notch (N) RNAi #1	*TinD-GAL4 UAS-Dcr-2/$N^{HMS00001}$*	0.87 µg/µl	2.17	2.65
Notch (N) RNAi #2	*TinD-GAL4 UAS-Dcr-2/$N^{HMS00009}$*	0.67 µg/µl	2.17	2.5
Suppressor of Hairless [Su(H)] RNAi #1	*$Su(H)^{HMS05748}$/+; TinD-GAL4 UAS-Dcr-2/+*	1.12 µg/µl	2.17	2.56
Suppressor of Hairless [Su(H)] RNAi #2	*TinD-GAL4 UAS-Dcr-2/$Su(H)^{HM05110}$*	1.06 µg/µl	2.14	2.44
groucho (gro) RNAi	*$gro^{KK108953}$/+; TinD-GAL4 UAS-Dcr-2/+*	1.53 µg/µl	2.21	2.63
Hairless (H) RNAi	*H^{GD1458}/+; TinD-GAL4 UAS-Dcr-2/+*	1.86 µg/µl	2.14	2.40
C-terminal Binding Protein (CtBP) RNAi	*$CtBP^{KK108401}$/+; TinD-GAL4 UAS-Dcr-2/+*	1.80 µg/µl	2.22	2.69

Table 1. Genotypes of embryos used as controls and for RNAi knockdowns of specific genes. The concentration and quality of total RNA obtained from these genotypes is listed.

cDNA synthesis for Reverse Transcription quantitative PCR (RT-qPCR)

cDNA was prepared with the SuperScript^{TM} IV VILO^{TM} Master Mix with ezDNase^{TM} Kit (ThermoFisher) using 2.5 µg of total RNA in 20 µl reactions according to the manufacturer's recommendations including the ezDNase^{TM} pre-treatment to remove genomic DNA. To verify efficient digestion of genomic DNA from the total RNA, an additional 2.5 µg of total RNA was also processed using the SuperScript^{TM} IV VILO^{TM} No RT Control Master Mix with ezDNase^{TM} pre-treatment. The No RT Control Master Mix was provided in the kit and contains all of the RT reaction components except for the reverse transcriptase (RT) enzyme. Both the RT+ and No RT Control 20 µl cDNA synthesis reactions were diluted 1:10 to a final volume of 200 µl using RNase/DNase-Free water such that they contained the original total RNA at a final concentration of 12.5 ng/µl.

Reverse Transcription quantitative PCR (RT-qPCR)

RT-qPCR was used to quantitate relative transcript expression levels between the different genotypes using the PowerUp^{TM} SYBR^{TM} Green Master Mix (ThermoFisher) in an Applied Biosystems^{TM} QuantStudio^{TM} 3 Real-Time PCR System (ThermoFisher). 10 µl qPCR reactions containing 2 µl (25 ng) of the reverse-transcribed total RNA, 1 pmol each of the relevant forward and reverse primers (Table 2), and 5 µl of the 2X master mix PCR reagent were utilized. qPCR reactions were performed in technical triplicate evaluating C_q reproducibility for each condition that was amplified. The standard deviation of the C_q varied between 0.005 to 0.389 for the technical replicates for each sample, thereby demonstrating C_q reproducibility well below a standard deviation of 0.5 C_q.

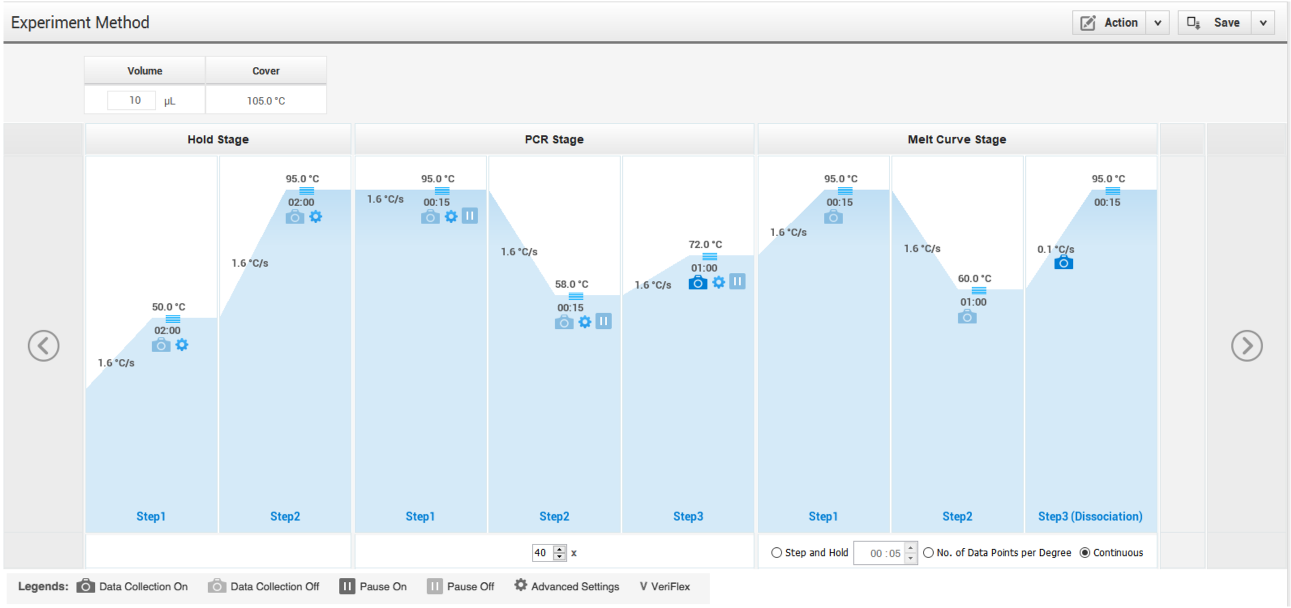

Fig 1. Standard cycling method used for RT-qPCR.

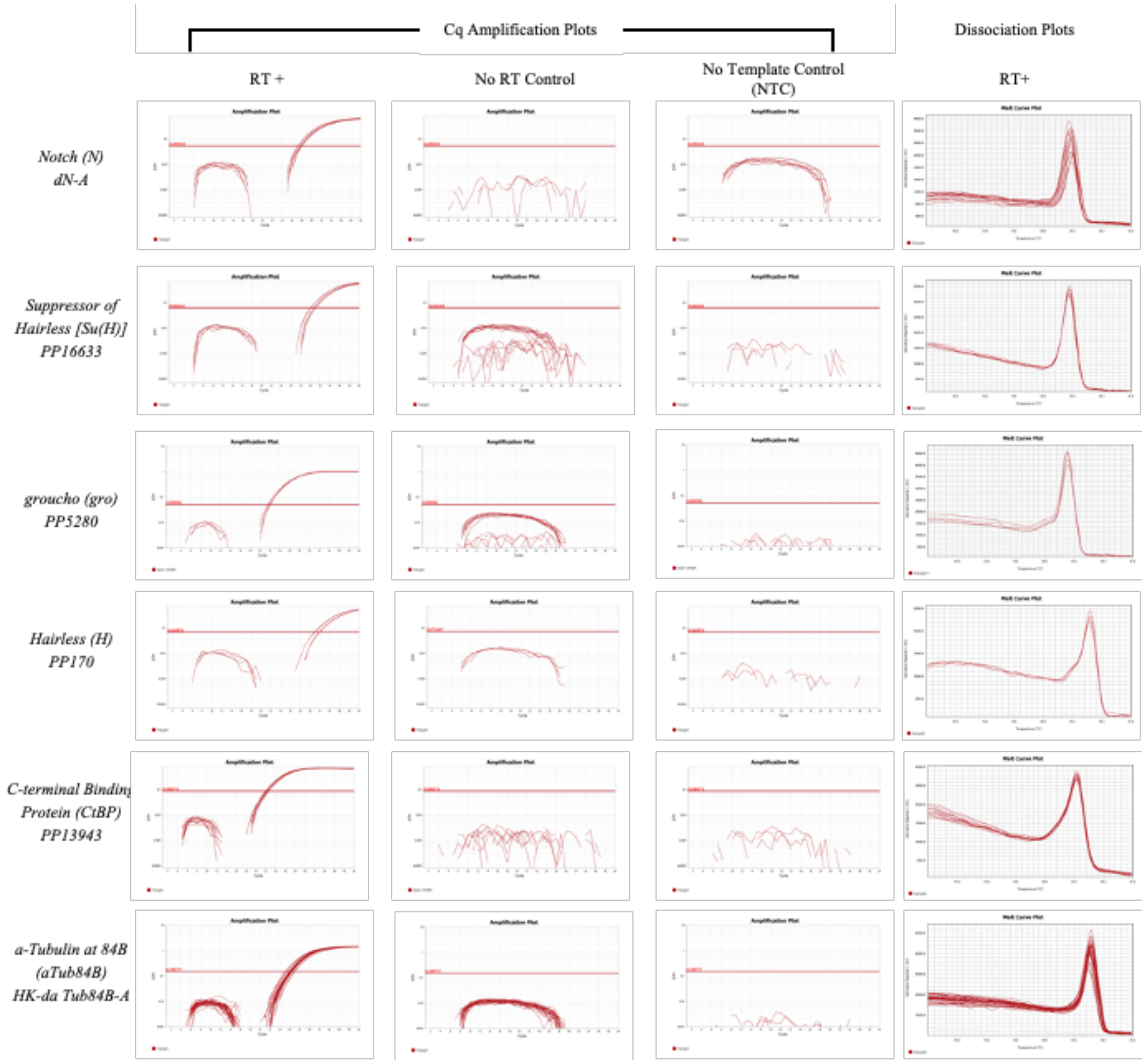

Fig 2. RT-qPCR C_q amplification plots and dissociation plots. Leftmost three columns of plots: C_q amplification plots for RT+, No RT control, and no template control (NTC) reactions for each of the primer sets investigated. All No RT Control C_q amplification plots remained below threshold, thereby demonstrating efficient removal of genomic DNA in the total RNA isolation procedure. All NTC reactions for each primer set also remained below threshold, thereby indicating an absence of amplification and thus no template contamination. Rightmost plot column: Dissociation (melt curve) plots of each primer set identifies a single amplification product in the RT+ reactions.

Gene	Primer name	Primer sequence 5′ to 3′′	Amplicon size
Notch (N)	dN-A-Forward<br>dN-A-Reverse	GAACGGCAATCCGAACAC<br>TTGATCATATCCAGACCATTGC	108
Suppressor of Hairless [Su(H)]	PP16633-Forward<br>PP16633-Reverse	CTTGCTGCCGGGTCCTTAC<br>CTCGCGCATGTACTTCTCCA	79
groucho (gro)	PP5280-Forward<br>PP5280-Reverse	CCTCAGGGTCCAATCAAGTTC<br>CACGTTAAGGCCATAGGACATC	180
Hairless (H)	PP170-Forward<br>PP170-Reverse	CCTCAAATGGCTTTTTGCCG<br>AAGGAACTGTAGTCCAGGACG	148
C-terminal Binding Protein (CtBP)	PP13943-Forward<br>PP13943-Reverse	GACTGGGCCGCATTGGTAG<br>AGGGATCGTAGAAGATGACGTT	75
α-Tubulin at 84B (αTub84B)	HK-da Tub84B-A-Forward<br>HK-da Tub84B-A-Reverse	ACGCTCTCTGAGTCAGACCT<br>CCAGCCTGACCAACATGGAT	130

Table 2. Gene-specific primers for RT-qPCR.

QuantStudio™ Design and Analysis Software VERSION 1.4.3 was used to run the experimental method, analyze, and determine the C_q values. The manufacturer's standard cycling method consisting of a UDG activation step, DNA polymerase activation step, PCR amplification stage, and dissociation curve stage was used for all qPCR reactions (Fig 1). The qPCR experimental parameters utilized the standard curve experimental type setting and determined the C_q value using both the Auto-Threshold and Auto-Baseline analysis functions. The qPCR analysis results were exported to Microsoft™ Excel™ for relative gene expression quantification. Relative gene expression was calculated using the $2^{-\Delta\Delta C_T}$ method [1]. *α-Tubulin at 84B* (*αTub84B*) was used as an endogenous reference gene to normalize targets because its primer set demonstrated a low C_q variance between all samples of 26.0 ± 0.51 (Mean C_q ± Standard Deviation) [2, 3].

At least three No Template Control (NTC) qPCR reactions for each primer set were included for each experiment, and they all demonstrated an absence of amplification and thus no template contamination (Fig 2). Similarly, the No RT Control qPCR reactions undertaken for each condition also exhibited no amplification and therefore efficient digestion and no carryover of genomic DNA (Fig 2).

Results

RT-qPCR analysis shows a decrease in *Notch* (*N*), *Suppressor of Hairless* [*Su(H)*], *groucho* (*gro*), *Hairless* (*H*), and *C-terminal Binding Protein* (*CtBP*) gene expression levels in the relevant RNAi knockdown embryos compared to the control embryos (Fig 3; Table 3). Note that the fact that considerable reduction in gene product levels of the relevant genes is detected with knockdowns while using total RNA from whole embryos despite the knockdown occurring in only a minute subset of embryonic cells (the cardiac mesoderm) indicates that the RNAi-induced knockdown in the cardiac mesoderm is quite severe.

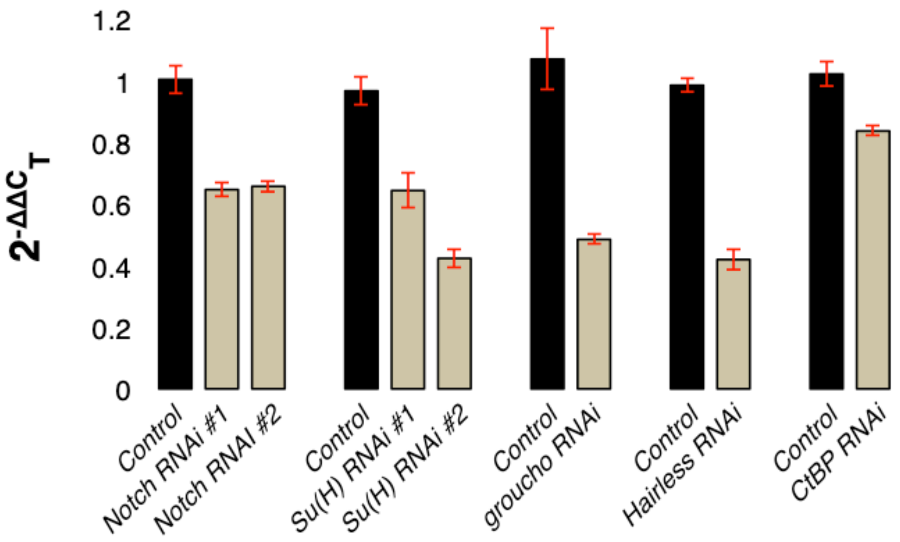

Fig 3. Relative *Notch*, *Su(H)*, *groucho*, *Hairless*, and *CtBP* gene expression levels, respectively, in appropriate RNAi knockdown embryos compared to control embryos calculated using the $2^{-\Delta\Delta C}{}_T$ method. Complete genotypes of control and RNAi knockdown embryos are listed in Table 1 and sequences of the primer pairs used are presented in Table 2. Bars indicate standard deviation.

Additionally, in the case of two knockdowns (that of *Su(H)* and *CtBP*) that were examined further, driving the RNAi constructs throughout the entire mesoderm with both *twist-GAL4* and *Mef2-GAL4* drivers produced relatively stronger knockdowns of those genes than those produced by driving the constructs solely in the smaller cardiogenic subset of the mesoderm by the cardiac mesoderm-specific *TinD-GAL4* driver (Fig 4; Table 4).

Finally, in order to examine the possibility of non-target effects in the RNAi-induced knockdown experiments, we assessed whether the *CtBP* RNAi knockdown also reduced the gene expression levels of *Su(H)*, *gro*, and *H* in addition to that of *CtBP* itself. Using appropriate primer sets, we observed that expression levels were maintained at near normal levels in the *CtBP* RNAi embryos and exhibited no reduction compared to those in the control embryos (Fig 5; Table 5), suggesting that there were no off-target effects in our RNAi experiments.

Gene	Primer Pair	Embryo	$2^{-\Delta\Delta C_T}$	SD
Notch	dN-A	Control	1.004	0.045
		Notch (N) RNAi #1	0.647	0.021
		Notch (N) RNAi #2	0.657	0.017
Su(H)	PP16633	Control	0.967	0.047
		Suppressor of Hairless [Su(H)] RNAi #1	0.644	0.055
		Suppressor of Hairless [Su(H)] RNAi #2	0.423	0.029
groucho	PP5280	Control	1.070	0.099
		groucho (gro) RNAi	0.485	0.016
Hairless	PP170	Control	0.985	0.021
		Hairless (H) RNAi	0.420	0.033
CtBP	PP13943	Control	1.022	0.039
		C-terminal Binding Protein (CtBP) RNAi	0.837	0.016

Table 3. Relative *Notch*, *Su(H)*, *groucho*, *Hairless*, and *CtBP* gene expression levels, respectively, in appropriate RNAi knockdown embryos compared to control embryos calculated using the $2^{-\Delta\Delta C_T}$ method. Complete genotypes of control and RNAi knockdown embryos are listed in Table 1 and sequences of the primer pairs used are presented in Table 2.

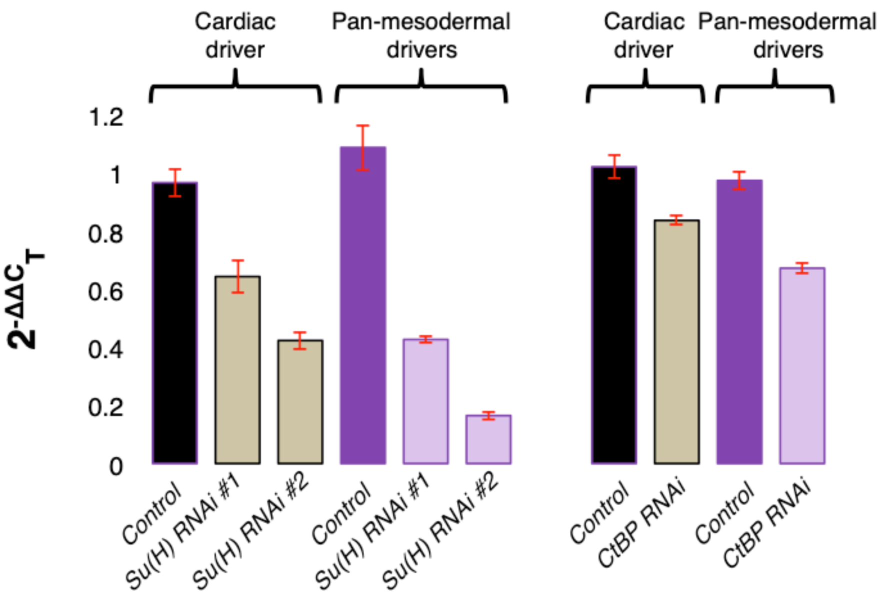

Fig 4. Relative knockdown of *Su(H)* and *CtBP* gene expression levels when the RNAi hairpin construct is driven by cardiac or pan-mesodermal drivers. Bars indicate standard deviation.

Gene	Primer Pair	Driver	Embryo	$2^{-\Delta\Delta C_T}$	SD
Su(H)	PP16633	Cardiac mesodermal (*TinD-GAL4*)	Control	0.967	0.047
			Suppressor of Hairless [Su(H)] RNAi #1	0.644	0.055
			Suppressor of Hairless [Su(H)] RNAi #2	0.423	0.029
		Pan-mesodermal (*twist-GAL4; Mef2-GAL4*)	Control	1.088	0.078
			Suppressor of Hairless [Su(H)] RNAi #1	0.427	0.010
			Suppressor of Hairless [Su(H)] RNAi #2	0.165	0.013
CtBP	PP13943	Cardiac mesodermal (*TinD-GAL4*)	Control	1.022	0.039
			C-terminal Binding Protein (CtBP) RNAi	0.837	0.016
		Pan-mesodermal (*twist-GAL4; Mef2-GAL4*)	Control	0.974	0.030
			C-terminal Binding Protein (CtBP) RNAi	0.673	0.017

Table 4. Relative knockdown of *Su(H)* and *CtBP* gene expression levels when the RNAi hairpin construct is driven by cardiac or pan-mesodermal drivers.

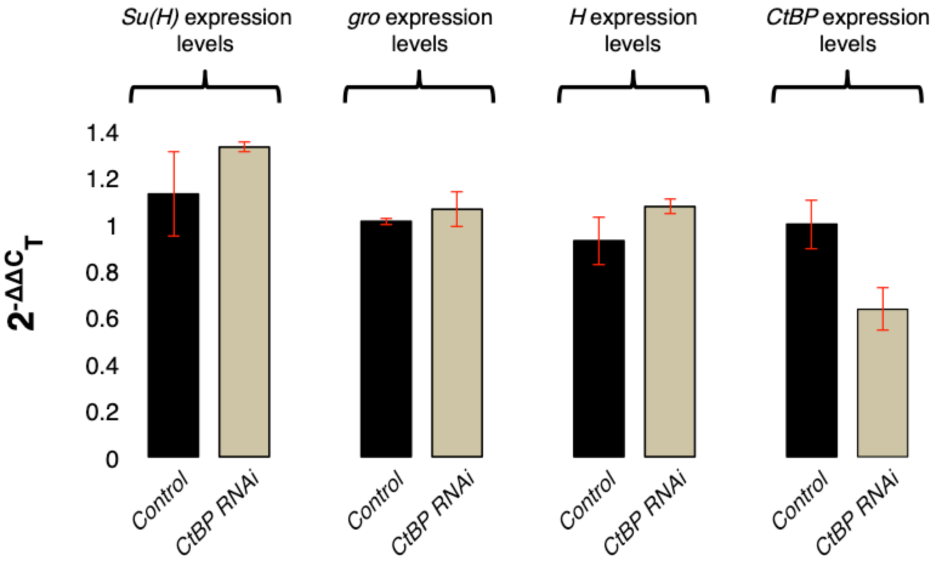

Fig 5. Relative *Su(H)*, *groucho*, *Hairless*, and *CtBP* gene expression levels, respectively, in the *CtBP* RNAi knockdown embryos compared to control embryos calculated using the $2^{-\Delta\Delta C_T}$ method. Bars indicate standard deviation. Note that *Su(H)*, *groucho*, and *Hairless* expression levels are maintained at near normal levels in the *CtBP* RNAi embryos and exhibit no reduction compared to those in the control embryos.

Gene	Primer Pair	Embryo	$2^{-\Delta\Delta C_T}$	SD
Su(H)	PP16633	Control	1.128	0.180
		C-terminal Binding Protein (CtBP) RNAi	1.329	0.022
groucho	PP5280	Control	1.010	0.015
		C-terminal Binding Protein (CtBP) RNAi	1.062	0.074
Hairless	PP170	Control	0.927	0.103
		C-terminal Binding Protein (CtBP) RNAi	1.074	0.032
CtBP	PP13943	Control	0.998	0.1.05
		C-terminal Binding Protein (CtBP) RNAi	0.633	0.092

Table 5. Relative *Su(H)*, *groucho*, *Hairless*, and *CtBP* gene expression levels, respectively, in the *CtBP* RNAi knockdown embryos compared to control embryos calculated using the $2^{-\Delta\Delta C_T}$ method.

References

1. Livak KJ, Schmittgen TD. Analysis of relative gene expression data using real-time quantitative PCR and the 2(-Delta Delta C(T)) Method. Methods. 2001;25(4):402-8. doi: 10.1006/meth.2001.1262. PubMed PMID: 11846609.

2. Ling D, Salvaterra PM. Robust RT-qPCR data normalization: validation and selection of internal reference genes during post-experimental data analysis. PLoS One. 2011;6(3):e17762. doi: 10.1371/journal.pone.0017762. PubMed PMID: 21423626; PubMed Central PMCID: PMC3058000.

3. Bustin SA, Benes V, Garson JA, Hellemans J, Huggett J, Kubista M, et al. The MIQE guidelines: minimum information for publication of quantitative real-time PCR experiments. Clin Chem. 2009;55(4):611-22. Epub 2009/02/28. doi: 10.1373/clinchem.2008.112797. PubMed PMID: 19246619.
