# Supplementary material for: Three distinct mechanisms, Notch instructive, permissive, and independent, regulate the expression of two different pericardial genes to specify cardiac cell subtypes
Source: PLoS One. 2020 Oct 27;15(10):e0241191. doi: 10.1371/journal.pone.0241191 (PMC7591092; doi:10.1371/journal.pone.0241191)
Supplement: S6 File — The complete sequence of this cloning vector, also available from GenBank with the accession number MT747949, is provided in GenBank flat file format. (PDF) [file pone.0241191.s011.pdf]

```
LOCUS       MT747949               11657 bp    DNA     circular SYN 17-JUL-2020
DEFINITION  Cloning vector pWattB-nlacZ, complete sequence.
ACCESSION   MT747949
VERSION     MT747949
KEYWORDS    .
SOURCE      Cloning vector pWattB-nlacZ
  ORGANISM  Cloning vector pWattB-nlacZ
            other sequences; artificial sequences; vectors.
REFERENCE   1  (bases 1 to 11657)
  AUTHORS   Ahmad SM, Bhattacharyya P, Jeffries N, Gisselbrecht SS and
            Michelson AM.
  TITLE     Two Forkhead transcription factors regulate cardiac progenitor
            specification by controlling the expression of receptors of the
            fibroblast growth factor and Wnt signaling pathways
  JOURNAL   Development 143 (2), 306-317 (2016)
   PUBMED   26657774
REFERENCE   2  (bases 1 to 11657)
  AUTHORS   Panta,M., Kump,A.J., Dalloul,J.M., Schwab,K.R. and Ahmad,S.M.
  TITLE     Three distinct mechanisms, Notch instructive, permissive, and
            independent, regulate the expression of two different pericardial
            genes to specify cardiac cell subtypes
  JOURNAL   Unpublished
REFERENCE   3  (bases 1 to 11657)
  AUTHORS   Ahmad,S.M.
  TITLE     Direct Submission
  JOURNAL   Submitted (12-JUL-2020) Department of Biology, Indiana State
            University, Science Building, Room 287H, 600 Chestnut St., Terre
            Haute, IN 47809, USA
COMMENT     ##Assembly-Data-START##
            Assembly Method       :: FlyE v. 2020-03-20
            Sequencing Technology :: PacBio
            ##Assembly-Data-END##
FEATURES             Location/Qualifiers
     source          1..11657
                     /organism="Cloning vector pWattB-nlacZ"
                     /mol_type="other DNA"
                     /db_xref="taxon:2754839"
     misc_recomb     89..373
                     /note="phiC31 attB site; derived from Streptomyces
                     lividans"
     gene            complement(1423..2283)
                     /gene="AmpR"
     CDS             complement(1423..2283)
                     /gene="AmpR"
                     /note="confers resistance to ampicillin, carbenicillin,
                     and related antibiotics"
                     /codon_start=1
                     /transl_table=11
                     /product="beta-lactamase"
                     /protein_id="QLI61520"
                     /translation="MSIQHFRVALIPFFAAFCLPVFAHPETLVKVKDAEDQLGARVGY
                     IELDLNSGKILESFRPEERFPMMSTFKVLLCGAVLSRIDAGQEQLGRRIHYSQNDLVE
                     YSPVTEKHLTDGMTVRELCSAAITMSDNTAANLLLTTIGGPKELTAFLHNMGDHVTRL
                     DRWEPELNEAIPNDERDTTMPVAMATTLRKLLTGELLTLASRQQLIDWMEADKVAGPL
                     LRSALPAGWFIADKSGAGERGSRGIIAALGPDGKPSRIVVIYTTGSQATMDERNRQIA
                     EIGASLIKHW"
     gene            complement(2574..5446)
```

```
                     /gene="mini-white"
     CDS             complement(join(2574..3188,3259..3390,3594..3909,
                     3971..4625,4700..4973,5375..5446))
                     /gene="mini-white"
                     /note="derived from Drosophila; lacks part of the first
                     intron"
                     /codon_start=1
                     /transl_table=11
                     /product="white"
                     /protein_id="QLI61519"
                     /translation="MGQEDQELLIRGGSKHPSAEHLNNGDSGAASQSCINQGFGQAKN
                     YGTLRPPSPPEDSGSGSGQLAENLTYAWHNMDIFGAVNQPGSGWRQLVNRTRGLFCNE
                     RHIPAPRKHLLKNVCGVAYPGELLAVMGSSGAGKTTLLNALAFRSPQGIQVSPSGMRL
                     LNGQPVDAKEMQARCAYVQQDDLFIGSLTAREHLIFQAMVRMPRHLTYRQRVARVDQV
                     IQELSLSKCQHTIIGVPGRVKGLSGGERKRLAFASEALTDPPLLICDEPTSGLDSFTA
                     HSVVQVLKKLSQKGKTVILTIHQPSSELFELFDKILLMAEGRVAFLGTPSEAVDFFSY
                     VGAQCPTNYNPADFYVQVLAVVPGREIESRDRIAKICDNFAISKVARDMEQLLATKNL
                     EKPLEQPENGYTYKATWFMQFRAVLWRSWLSVLKEPLLVKVRLIQTTMVAILIGLIFL
                     GQQLTQVGVMNINGAIFLFLTNMTFQNVFATINVFTSELPVFMREARSRLYRCDTYFL
                     GKTIAELPLFLTVPLVFTAIAYPMIGLRAGVLHFFNCLALVTLVANVSTSFGYLISCA
                     SSSTSMALSVGPPVIIPFLLFGGFFLNSGSVPVYLKWLSYLSWFRYANEGLLINQWAD
                     VEPGEISCTSSNTTCPSSGKVILETLNFSAADLPLDYVGLAILIVSFRVLAYLALRLR
                     ARRKE"
     regulatory      6097..6491
                     /regulatory_class="insulator"
                     /note="gypsy"
     misc_feature    6496..6586
                     /note="multiple cloning site"
     regulatory      6596..6687
                     /regulatory_class="TATA_box"
                     /note="Hsp27"
     gene            6735..9833
                     /gene="lacZ"
     CDS             6735..9833
                     /gene="lacZ"
                     /note="derived from Escherichia coli; contains nuclear
                     localization signal"
                     /codon_start=1
                     /transl_table=11
                     /product="beta-galactosidase"
                     /protein_id="QLI61521"
                     /translation="MGDPTPPKKKRKVEDDPVVLQRRDWENPGVTQLNRLAAHPPFAS
                     WRNSEEARTDRPSQQLRSLNGEWRFAWFPAPEAVPESWLECDLPEADTVVVPSNWQMH
                     GYDAPIYTNVTYPITVNPPFVPTENPTGCYSLTFNVDESWLQEGQTRIIFDGVNSAFH
                     LWCNGRWVGYGQDSRLPSEFDLSAFLRAGENRLAVMVLRWSDGSYLEDQDMWRMSGIF
                     RDVSLLHKPTTQISDFHVATRFNDDFSRAVLEAEVQMCGELRDYLRVTVSLWQGETQV
                     ASGTAPFGGEIIDERGGYADRVTLRLNVENPKLWSAEIPNLYRAVVELHTADGTLIEA
                     EACDVGFREVRIENGLLLLNGKPLLIRGVNRHEHHPLHGQVMDEQTMVQDILLMKQNN
                     FNAVRCSHYPNHPLWYTLCDRYGLYVVDEANIETHGMVPMNRLTDDPRWLPAMSERVT
                     RMVQRDRNHPSVIIWSLGNESGHGANHDALYRWIKSVDPSRPVQYEGGGADTTATDII
                     CPMYARVDEDQPFPAVPKWSIKKWLSLPGETRPLILCEYAHAMGNSLGGFAKYWQAFR
                     QYPRLQGGFVWDWVDQSLIKYDENGNPWSAYGGDFGDTPNDRQFCMNGLVFADRTPHP
                     ALTEAKHQQQFFQFRLSGQTIEVTSEYLFRHSDNELLHWMVALDGKPLASGEVPLDVA
                     PQGKQLIELPELPQPESAGQLWLTVRVVQPNATAWSEAGHISAWQQWRLAENLSVTLP
                     AASHAIPHLTTSEMDFCIELGNKRWQFNRQSGFLSQMWIGDKKQLLTPLRDQFTRAPL
                     DNDIGVSEATRIDPNAWVERWKAAGHYQAEAALLQCTADTLADAVLITTAHAWQHQGK
                     TLFISRKTYRIDGSGQMAITVDVEVASDTPHPARIGLNCQLAQVAERVNWLGLGPQEN
```

```
                     YPDRLTAACFDRWDLPLSDMYTPYVFPSENGLRCGTRELNYGPHQWRGDFQFNISRYS
                     QQQLMETSHRHLLHAEEGTWLNIDGFHMGIGGDDSWSPSVSAELQLSAGRYHYQLVWC
                     QK"
     regulatory      10987..11373
                     /regulatory_class="insulator"
                     /note="gypsy"
ORIGIN
        1 agcgtcaatg tccgccttca gttgcatttt gtcagcggtt tcgtgacgaa gctccaagcg
       61 gtttacgcca tcaattaaac acggccgcgt cgacgatgta ggtcacggtc tcgaagccgc
      121 ggtgcgggtg ccagggcgtg cccttgggct ccccgggcgc gtactccacc tcacccatct
      181 ggtccatcat gatgaacggg tcgaggtggc ggtagttgat cccggcgaac gcgcggcgca
      241 ccgggaagcc ctcgccctcg aaaccgctgg gcgcggtggt cacggtgagc acgggacgtg
      301 cgacggcgtc ggcgggtgcg gatacgcggg gcagcgtcag cgggttctcg acggtcacgg
      361 cgggcatgtc gactcgaggc cggtctccct atagtgagtc gtattaattt cgataagcca
      421 ggttaacctg cattaatgaa tcggccaacg cgcggggaga ggcggtttgc gtattgggcg
      481 ctcttccgct tcctcgctca ctgactcgct gcgctcggtc gttcggctgc ggcgagcggt
      541 atcagctcac tcaaaggcgg taatacggtt atccacagaa tcaggggata acgcaggaaa
      601 gaacatgtga gcaaaaggcc agcaaaaggc caggaaccgt aaaaaggccg cgttgctggc
      661 gtttttccat aggctccgcc cccctgacga gcatcacaaa aatcgacgct caagtcagag
      721 gtggcgaaac ccgacaggac tataaagata ccaggcgttt ccccctggaa gctccctcgt
      781 gcgctctcct gttccgaccc tgccgcttac cggatacctg tccgcctttc tcccttcggg
      841 aagcgtggcg ctttctcata gctcacgctg taggtatctc agttcggtgt aggtcgttcg
      901 ctccaagctg ggctgtgtgc acgaaccccc cgttcagccc gaccgctgcg ccttatccgg
      961 taactatcgt cttgagtcca acccggtaag acacgactta tcgccactgg cagcagccac
     1021 tggtaacagg attagcagag cgaggtatgt aggcggtgct acagagttct tgaagtggtg
     1081 gcctaactac ggctacacta gaagaacagt atttggtatc tgcgctctgc tgaagccagt
     1141 taccttcgga aaaagagttg gtagctcttg atccggcaaa caaaccaccg ctggtagcgg
     1201 tggttttttt gtttgcaagc agcagattac gcgcagaaaa aaaggatctc aagaagatcc
     1261 tttgatcttt tctacggggt ctgacgctca gtggaacgaa aactcacgtt aagggatttt
     1321 ggtcatgaga ttatcaaaaa ggatcttcac ctagatcctt ttaaattaaa aatgaagttt
     1381 taaatcaatc taaagtatat atgagtaaac ttggtctgac agttaccaat gcttaatcag
     1441 tgaggcacct atctcagcga tctgtctatt tcgttcatcc atagttgcct gactccccgt
     1501 cgtgtagata actacgatac gggagggctt accatctggc cccagtgctg caatgatacc
     1561 gcgagaccca cgctcaccgg ctccagattt atcagcaata aaccagccag ccggaagggc
     1621 cgagcgcaga agtggtcctg caactttatc cgcctccatc cagtctatta attgttgccg
     1681 ggaagctaga gtaagtagtt cgccagttaa tagtttgcgc aacgttgttg ccattgctac
     1741 aggcatcgtg gtgtcacgct cgtcgtttgg tatggcttca ttcagctccg gttcccaacg
     1801 atcaaggcga gttacatgat cccccatgtt gtgcaaaaaa gcggttagct ccttcggtcc
     1861 tccgatcgtt gtcagaagta agttggccgc agtgttatca ctcatggtta tggcagcact
     1921 gcataattct cttactgtca tgccatccgt aagatgcttt tctgtgactg gtgagtactc
     1981 aaccaagtca ttctgagaat agtgtatgcg gcgaccgagt tgctcttgcc cggcgtcaat
     2041 acgggataat accgcgccac atagcagaac tttaaaagtg ctcatcattg gaaaacgttc
     2101 ttcggggcga aaactctcaa ggatcttacc gctgttgaga tccagttcga tgtaacccac
     2161 tcgtgcaccc aactgatctt cagcatcttt tactttcacc agcgtttctg ggtgagcaaa
     2221 aacaggaagg caaaatgccg caaaaaaggg aataagggcg acacggaaat gttgaatact
     2281 catactcttc ctttttcaat attattgaag catttatcag ggttattgtc tcatgagcgg
     2341 atacatattt gaatgtattt agaaaaataa acaaataggg gttccgcgca catttccccg
     2401 aaaagtgcca cctgacgtct tcgaagggga aatacttgta ttctataggt catatcttgt
     2461 ttttattggc acaaatataa ttacattagc tttttgaggg ggcaataaac agtaaacacg
     2521 atggtaataa tggtaaaaaa aaaaaacaag cagttatttc ggatatatgt cggctactcc
     2581 ttgcgtcggg cccgaagtct tagagccaga tatgcgagca cccggaagct cacgatgaga
     2641 atggccagac ccacgtagtc cagcggcaga tcggcggcgg agaagttaag cgtctccagg
     2701 atgaccttgc ccgaactggg gcacgtggtg ttcgacgatg tgcagctaat ttcgcccggc
     2761 tccacgtccg cccattggtt aatcagcaga ccctcgttgg cgtaacggaa ccatgagagg
     2821 tacgacaacc atttgaggta tactggcacc gagcccgagt tcaagaagaa gccgccaaag
     2881 agcaggaatg gtatgataac cggcggaccc acagacagcg ccatcgaggt cgaggagctg
     2941 gcgcaggata ttagatatcc gaaggacgtt gacacattgg ccaccagagt gaccagcgcc
```

3001 aggcagttga agaagtgcag cactccggcc cgcagtccga tcatcggata ggcaatcgcc
3061 gtgaagacca gtggcactgt gagaaaaagc ggcaattcgg caatcgtttt gcccagaaag
3121 tatgtgtcac agcgataaag tcgacttcgg gcctccctca taaaaactgg cagctctgag
3181 gtgaacacct aaatcgaatc gattcattag aaagttagta aattattgaa atgcaaatgt
3241 attctaaaca tgacttacat ttatcgtggc aaagacgttt tgaaaggtca tgttggtcag
3301 gaagaggaag atggctccgt tgatattcat cacacccact tgcgtgagtt gttggcccaa
3361 aaagatgagg ccaatcaaga tggcaaccat ctgcaaatta aaatgttact cgcatctcat
3421 taatattcgc gagttaaatg aaatttattt atcttctgca aaactataaa ctatacatct
3481 cattgaaaaa aactaagaag ggtgtggaat caggcaattc tatctaaaat ctagcgaatt
3541 tgtttccaag aattgtaagc gttatatcat ttgtttccac tggaaccact caccgttgtc
3601 tgaataagtc gcacttttac gaggagtggt tccttgagca ccgacagcca ggatcgccac
3661 aggaccgccc ggaactgcat gaaccaggtg gccttgtagg tgtacccatt ctccggctgc
3721 tccagtggct tctccagatt tttggtggcc aacaactgct ccatatcccg ggctactttg
3781 ctaatggcaa aattgtcgca tatcttggcg atccgatcac gggactcgat ctcccgtccg
3841 ggcacaacgg ccaacacctg tacgtaaaag tccgccggat tgtagttggt aggacactgg
3901 gcacccacgc tggataggag ttgagatgtt atgtaatact agatacccctt aataaacaca
3961 tcgaactcac taggaaaaga agtcgacggc ttcgctggga gtgcccaaga aagctaccct
4021 gccctcggcc atcagaagga tcttgtcaaa gagctcaaac agctcggaag acggctgatg
4081 aatggtcagg atgacggtct tgcccttctg cgacagcttc ttcagcacct ggacgacgct
4141 gtgggcggta aaggagtcca gtccggaggt gggctcatcg cagatcagaa gcggcggatc
4201 ggttagagcc tcggaggcga atgccagacg cttcctttct ccgccggaca gacctttcac
4261 cctgccgggc acaccgatga tcgtgtgctg acatttgctg agcgaaagct cctggatcac
4321 ctgatccacg cgggccactc gctgccgata ggtcagatgt cgtggcatcc gcaccatggc
4381 ttggaaaatc aggtgttccc tggccgttag ggagccgata aagaggtcat cctgctggac
4441 ataggcgcac ctggcctgca tctccttggc gtccacaggt tggccattga gcagtcgcat
4501 cccggatggc gatacttgga tgccctgcgg cgatcgaaag gcaagggcat tcagcagggt
4561 cgtctttccg gcaccggaac tgcccatcac ggccaaaagt tcgcccggat aggccacgcc
4621 gcaaactgag tttcaaattg gtaattggac cctttattaa gatttcacac agatcagccg
4681 actgcgaata gaaactcacc gttcttgagc aaatgtttcc tgggcgccgg tatgtgtcgc
4741 tcgttgcaga atagtccgcg tgtccggttg accagctgcc gccatccgga gcccggctga
4801 ttgaccgccc caaagatgtc catattgtgc caggcatagg tgaggttctc ggctagttgg
4861 ccgctccctg aaccggagtc ctccggcgga ctgggtggcc ggagcgtgcc gtagtttttg
4921 gcctgcccga agccctggtt aatgcagctc tgcgaagccg ctccgctgtc accctgcaat
4981 gataggggat ctcaaatatc aactacaagc gttatgctca tctaaccccg aacaaaaagt
5041 accccgaagt atcctacgaa gtaggtttat acttttattt attttttgtg catctaggat
5101 cagcttaaaa tatctggttg ttatattttt tgtaaaaaag aatatagtcg aaaatgaatg
5161 cctttagatg tcttgatcat gatatgatct caaaaattgt cttatatagc gagaacagct
5221 accagaataa tctgtttcgt gtcactattt gtttgtgcaa ttgcggtttg ggatttttgt
5281 gggtcgcagt tctcacgccg cagacaattt gatgttgcaa tcgcagttcc tatagatcaa
5341 gtgaacttaa gatgtatgca catgtactac tcacattgtt cagatgctcg gcagatgggt
5401 gtttgctgcc tccgcgaatt aatagctcct gatcctcttg gcccattgcc gggatttttc
5461 acactttccc ctgcttaccc acccaaaacc aatcaccacc ccaatcactc aaaaaacaaa
5521 caaaaataag aagcgagagg agttttggca cagcactttg tgtttaattg atggcgtaaa
5581 ccgcttggag cttcgtcacg aaaccgctga caaaatgcaa ctgaaggcgg acattgacgc
5641 tacgtaacgc tacaaacggt ggcgaaagag atagcggacg cagcggcgaa agagacggcg
5701 atatttctgt ggacagagaa ggaggcaaac agcccggacg tctaagaaac cattattatc
5761 atgacattaa cctataaaaa taggcgtatc acgaggccct ttcgtctcgc gcgtttcggt
5821 gatgacggtg aaaacctctg acacatgcag ctcccggaga cggtcacagc ttgtctgtaa
5881 gcggatgccg ggagcagaca agcccgtcag ggcgcgtcag cgggtgttgg cgggtgtcgg
5941 ggctggctta actatgcggc atcagagcag attgtactga gagtgcacca tatggacata
6001 ttgtcgttag aacgcggcta caattaatac ataaccttat gtatcataca catacgattt
6061 aggtgacact atagaaccag atcagcttgg ctgcatcacg taataagtgt gcgttgaatt
6121 tattcgcaaa aacattgcat attttcggca aagtaaaatt ttgttgcata ccttatcaaa
6181 aaataagtgc tgcatacttt ttagagaaac caaataattt tttattgcat acccgttttt
6241 aataaaatac attgcatacc ctcttttaat aaaaaatatt gcatactttg acgaaacaaa
6301 ttttcgttgc atacccaata aaagattatt atattgcata cccgttttta ataaaataca
6361 ttgcataccc tcttttaata aaaaatattg catacgttga cgaaacaaat tttcgttgca

6421 tacccaataa aagattatta tattgcatac cttttcttgc cataccattt agccgatcaa
6481 ttgtgctcgg caacagcatg ctgcagcaga tctggtctag agcccgggcg aattcgccgg
6541 cgcgccggta ccccgcggcc gctagcggat ccgcggccgc tacgtatcta gagtcgagcc
6601 cagcgtcagt ataaaagccg gcgtcaacgt cgaccgagca cagtctaaac tgaaaaattg
6661 aaggcaaacg ttgaagcaaa cttcgctaaa aaaaaattcg aaaaggcaaa aaaaattcct
6721 ttgtctagac caccatggga gatcccaccc cacccaagaa gaagcgcaag gtggaggacg
6781 atcccgtcgt tttacaacgt cgtgactggg aaaaccctgg cgttacccaa cttaatcgcc
6841 ttgcagcaca tccccctttc gccagctggc gtaatagcga agaggcccgc accgatcgcc
6901 cttcccaaca gttgcgcagc ctgaatggcg aatggcgctt tgcctggttt ccggcaccag
6961 aagcggtgcc ggaaagctgg ctggagtgcg atcttcctga ggccgatact gtcgtcgtcc
7021 cctcaaactg gcagatgcac ggttacgatg cgcccatcta caccaacgtg acctatccca
7081 ttacggtcaa tccgccgttt gttcccacgg agaatccgac gggttgttac tcgctcacat
7141 ttaatgttga tgaaagctgg ctacaggaag gccagacgcg aattattttt gatggcgtta
7201 actcggcgtt tcatctgtgg tgcaacgggc gctgggtcgg ttacggccag gacagtcgtt
7261 tgccgtctga atttgacctg agcgcatttt tacgcgccgg agaaaaccgc ctcgcggtga
7321 tggtgctgcg ctggagtgac ggcagttatc tggaagatca ggatatgtgg cggatgagcg
7381 gcattttccg tgacgtctcg ttgctgcata aaccgactac acaaatcagc gatttccatg
7441 ttgccactcg ctttaatgat gatttcagcc gcgctgtact ggaggctgaa gttcagatgt
7501 gcggcgagtt gcgtgactac ctacgggtaa cagtttcttt atggcagggt gaaacgcagg
7561 tcgccagcgg caccgcgcct ttcggcggtg aaattatcga tgagcgtggt ggttatgccg
7621 atcgcgtcac actacgtctg aacgtcgaaa acccgaaact gtggagcgcc gaaatcccga
7681 atctctatcg tgcggtggtt gaactgcaca ccgccgacgg cacgctgatt gaagcagaag
7741 cctgcgatgt cggtttccgc gaggtgcgga ttgaaaatgg tctgctgctg ctgaacggca
7801 agccgttgct gattcgaggc gttaaccgtc acgagcatca tcctctgcat ggtcaggtca
7861 tggatgagca gacgatggtg caggatatcc tgctgatgaa gcagaacaac tttaacgccg
7921 tgcgctgttc gcattatccg aaccatccgc tgtggtacac gctgtgcgac cgctacggcc
7981 tgtatgtggt ggatgaagcc aatattgaaa cccacggcat ggtgccaatg aatcgtctga
8041 ccgatgatcc gcgctggcta ccggcgatga gcgaacgcgt aacgcgaatg gtgcagcgcg
8101 atcgtaatca cccgagtgtg atcatctggt cgctggggaa tgaatcaggc cacggcgcta
8161 atcacgacgc gctgtatcgc tggatcaaat ctgtcgatcc ttcccgcccg gtgcagtatg
8221 aaggcggcgg agccgacacc acggccaccg atattatttg cccgatgtac gcgcgcgtgg
8281 atgaagacca gcccttcccg gctgtgccga aatggtccat caaaaaatgg ctttcgctac
8341 ctggagagac gcgcccgctg atcctttgcg aatacgccca cgcgatgggt aacagtcttg
8401 gcggtttcgc taaatactgg caggcgtttc gtcagtatcc ccgtttacag ggcggcttcg
8461 tctgggactg ggtggatcag tcgctgatta aatatgatga aaacggcaac ccgtggtcgg
8521 cttacggcgg tgattttggc gatacgccga acgatcgcca gttctgtatg aacggtctgg
8581 tctttgccga ccgcacgccg catccagcgc tgacggaagc aaaacaccag cagcagtttt
8641 tccagttccg tttatccggg caaaccatcg aagtgaccag cgaatacctg ttccgtcata
8701 gcgataacga gctcctgcac tggatggtgg cgctggatgg taagccgctg gcaagcggtg
8761 aagtgcctct ggatgtcgct ccacaaggta aacagttgat tgaactgcct gaactaccgc
8821 agccggagag cgccgggcaa ctctggctca cagtacgcgt agtgcaaccg aacgcgaccg
8881 catggtcaga agccgggcac atcagcgcct ggcagcagtg gcgtctggcg gaaaacctca
8941 gtgtgacgct ccccgccgcg tcccacgcca tcccgcatct gaccaccagc gaaatggatt
9001 tttgcatcga gctgggtaat aagcgttggc aatttaaccg ccagtcaggc tttctttcac
9061 agatgtggat tggcgataaa aaacaactgc tgacgccgct gcgcgatcag ttcacccgtg
9121 caccgctgga taacgacatt ggcgtaagtg aagcgacccg cattgaccct aacgcctggg
9181 tcgaacgctg gaaggcggcg ggccattacc aggccgaagc agcgttgttg cagtgcacgg
9241 cagatacact tgctgatgcg gtgctgatta cgaccgctca cgcgtggcag catcagggga
9301 aaaccttatt tatcagccgg aaaacctacc ggattgatgg tagtggtcaa atggcgatta
9361 ccgttgatgt tgaagtggcg agcgatacac cgcatccggc gcggattggc ctgaactgcc
9421 agctggcgca ggtagcagag cgggtaaact ggctcggatt agggccgcaa gaaaactatc
9481 ccgaccgcct tactgccgcc tgttttgacc gctgggatct gccattgtca gacatgtata
9541 ccccgtacgt cttcccgagc gaaaacggtc tgcgctgcgg gacgcgcgaa ttgaattatg
9601 gcccacacca gtggcgcggc gacttccagt tcaacatcag ccgctacagt caacagcaac
9661 tgatggaaac cagccatcgc catctgctgc acgcggaaga aggcacatgg ctgaatatcg
9721 acggtttcca tatggggatt ggtggcgacg actcctggag cccgtcagta tcggcggaat
9781 tacagctgag cgccggtcgc taccattacc agttggtctg gtgtcaaaaa taataataac

```
     9841 cgggcaggcc atgtctgccc gtatttcgcg taaggaaatc cattatgtac tatttaaaaa
     9901 acacaaactt ttggatgttc ggtttattct ttttctttta cttttttatc atgggagcct
     9961 acttcccgtt tttcccgatt tggctacatg acatcaacca tatcagcaaa agtgatacgg
    10021 gtattatttt tgccgctatt tctctgttct cgctattatt ccaaccgctg tttggtctgc
    10081 tttctgacaa actcggcctc gactctagct agaggatctt tgtgaaggaa ccttacttct
    10141 gtggtgtgac ataattggac aaactaccta cagagattta aagctctaag gtaaatataa
    10201 aatttttaag tgtataatgt gttaaactac tgattctaat tgtttgtgta ttttagattc
    10261 caacctatgg aactgatgaa tgggagcagt ggtggaatgc ctttaatgag gaaaacctgt
    10321 tttgctcaga agaaatgcca tctagtgatg atgaggctac tgctgactct caacattcta
    10381 ctcctccaaa aaagaagaga aaggtagaag accccaagga ctttccttca gaattgctaa
    10441 gtttttgag tcatgctgtg tttagtaata gaactcttgc ttgctttgct atttacacca
    10501 caaaggaaaa agctgcactg ctatacaaga aaattatgga aaaatattct gtaaccttta
    10561 taagtaggca taacagttat aatcataaca tactgttttt tcttactcca cacaggcata
    10621 gagtgtctgc tattaataac tatgctcaaa aattgtgtac ctttagcttt ttaatttgta
    10681 aaggggttaa taaggaatat ttgatgtata gtgccttgac tagagatcat aatcagccat
    10741 accacatttg tagaggtttt acttgcttta aaaaacctcc cacacctccc cctgaacctg
    10801 aaacataaaa tgaatgcaat tgttgttgtt aacttgttta ttgcagctta taatggttac
    10861 aaataaagca atagcatcac aaatttcaca aataaagcat ttttttcact gcattctagt
    10921 tgtggtttgt ccaaactcat caatgtatct tatcatgtct ggatcgggcg agctcgaatt
    10981 gactagtcac gtaataagtg tgcgttgaat ttattcgcaa aaacattgca tattttcggc
    11041 aaagtaaaat tttgttgcat accttatcaa aaaataagtg ctgcatactt tttagagaaa
    11101 ccaaataatt ttttattgca tacccgtttt taataaaata cattgcatac cctcttttaa
    11161 taaaaaatat tgcatacttt gacgaaacaa attttcgttg catacccaat aaaagattat
    11221 tatattgcat acccgttttt aataaaatac attgcatacc ctcttttaat aaaaaatatt
    11281 gcatacgttg acgaaacaaa ttttcgttgc atacccaata aaagattatt atattgcata
    11341 ccttttcttg ccataccatt tagccgatca attctagtat gtatgtaagt taataaaacc
    11401 catttttgcg gaaagtagat aaaaaaaaca tttttttttt ttactgcact ggatatcatt
    11461 gaacttatct gatcagtttt aaatttactt cgatccaagg gtatttgatg taccaggttc
    11521 tttcgattac ctctcactca aaatgacatt ccactcaaag tcagcgctgt ttgcctcctt
    11581 ctctgtccac agaaatatcg ccgtctcttt cgccgctgcg tccgctatct ctttcgccac
    11641 cgtttgtagc gttacgt
//
```
